# Crystal structures of the alkali aluminoboracites *A*_4_B_4_Al_3_O_12_Cl (*A* = Li, Na)

**DOI:** 10.1107/S2056989024000501

**Published:** 2024-01-26

**Authors:** Sho Yoshino, Hidechika Arima, Masanao Ishijima, Koichi Kajihara

**Affiliations:** a Department of Applied Chemistry for Environment, Graduate School of Urban Environmental Sciences, Tokyo Metropolitan University, 1-1 Minami-Osawa, Hachoji, Tokyo 192-0397, Japan; Vienna University of Technology, Austria

**Keywords:** alkali aluminoboracite, self-flux method, weak structure ordering, isotypism, crystal structure

## Abstract

Single crystals of alkali aluminoboracites, *A*_4_B_4_Al_3_O_12_Cl (*A* = Li, Na), were grown using the self-flux method, and their isotypic cubic crystal structures were determined by single-crystal X-ray diffraction. Na_4_B_4_Al_3_O_12_Cl is the first reported sodium boracite, and its lattice parameter [13.5904 (1) Å] is the largest among the boracites consisting of a cation–oxygen framework reported so far. For both crystals, structure models refined in the cubic space group *F*  3*c*, which assume that all cubic octant subcells in the unit cell are equivalent, converged with *R*1 factors of ∼0.03. However, the presence of weak *hhl* reflections with odd *h* and *l* values indicates that refinements in the space group *F*23, which presume a checkerboard-like ordering of two types of subcells with slightly different atomic positions, are more appropriate.

## Abstract 2

Single crystals of cubic alkali aluminoboracites *A*_4_B_4_Al_3_O_12_Cl (*A* = Li, Na) were grown from a self-flux and their crystal structures were determined and compared in two space group types, *F* 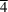 3*c* and *F*23. Na_4_B_4_Al_3_O_12_Cl is the first sodium boracite and its lattice parameter is the largest among boracites with a cation–oxygen framework.

## 1. Chemical context

Boracite is originally known as a mineral with the formula Mg_3_B_7_O_13_Cl. The name boracite also refers to borate compounds with the general formula *M*_3_B_7_O_13_ *X*, consisting of a negatively charged B–O framework, extraframework divalent cations *M*, such as Mg^2+^, Cr^2+^, Mn^2+^, Fe^2+^, Co^2+^, Ni^2+^, Cu^2+^, Zn^2+^ and Cd^2+^, and extraframework anions *X*, such as Cl^−^, Br^−^, I^−^ and S^2−^ (Schmid, 1965 ▸; Nelmes, 1974 ▸). The extraframework cations can also be an alkali ion, but only lithium variants Li_4–*x*_B_7_O_12+*x*/2_ *X*, Li_4_B_7–3*x*_Al_3*x*_O_12_ *X* and Li_4_B_7–3*x*_Ga_3*x*_O_12_Cl (*X* = Cl, Br; *x* = 0–1) have been reported to date (Levasseur *et al.*, 1971 ▸; Réau *et al.*, 1976 ▸; Jeitschko *et al.*, 1977 ▸; Calès *et al.*, 1977 ▸; Sorokin, 2015 ▸; Tezuka *et al.*, 2017 ▸; Kajihara *et al.*, 2017 ▸; Katsumata *et al.*, 2022 ▸). The latter two compounds (Kajihara *et al.*, 2017 ▸; Katsumata *et al.*, 2022 ▸) are the first examples of boracites containing framework cations other than B^3+^ ions. The lithium boracites are lithium-ion conducting, and their dc Li^+^ ion conductivity can be increased to ∼10^−5^ S cm^−1^ at room temperature in glass-ceramics con­sisting mainly of Li_4_B_4_ *M*_3_O_12_Cl (*M* = Al, Ga; Kajihara *et al.*, 2017 ▸). In addition, Li_4_B_4_Al_3_O_12_Cl is stable in contact with Li metal and is water-resistant; solid-state cells consisting of an Li_4_B_4_Al_3_O_12_Cl-based glass-ceramic solid electrolyte, an LiCoO_2_-based composite cathode containing an ionic liquid and an Li–Au alloy anode worked successfully (Saito *et al.*, 2021 ▸). More recently, a thio­boracite, Li_6_B_7_S_13_I, a sulfide variant of lithium boracite with room-temperature Li^+^ ion conductivity of ∼5 × 10^−4^ S cm^−1^ has been reported (Kaup *et al.*, 2021 ▸). However, single crystals of Li_4_B_4_Al_3_O_12_Cl have not yet been grown, and the preliminary crystal structure analysis of Li_4_B_4_Al_3_O_12_Cl (Kajihara *et al.*, 2017 ▸; Katsumata *et al.*, 2022 ▸) was incomplete. In addition, boracites containing alkali ions other than Li^+^ have not been reported. Furthermore, a rhombohedral distortion of the unit cell of single-crystalline Li_4_B_7_O_12_Cl was experimentally observed at room temperature (Jeitschko *et al.*, 1977 ▸) and was recently theoretically confirmed (Li & Holzwarth, 2022 ▸), raising the question whether similar unit-cell distortions occur in other alkali boracites.

In the present study, we report the growth of single crystals of *A*_4_B_4_Al_3_O_12_Cl (*A* = Li, Na) using the self-flux method and their structural characterization by single-crystal X-ray diffraction (XRD).

## 2. Structural commentary

The crystallites of Li_4_B_4_Al_3_O_12_Cl exhibit complete extinction under cross-polarized light, supporting cubic symmetry. Hence, the unit cell of Li_4_B_4_Al_3_O_12_Cl is not distorted. At first, the crystal structure was refined in the noncentrosymmetric cubic space group *F* 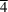 3*c* following the model from the Rietveld refinement of powder X-ray diffraction data (Kajihara *et al.*, 2017 ▸; Katsumata *et al.*, 2022 ▸). The occupancy (*g*) of Cl1 converged to the upper bound [*g*(Cl1) = 1] and was fixed at this value. The reliability factor *R*1 converged to 0.031, and the refinement results agreed well with those derived from the powder samples (Kajihara *et al.*, 2017 ▸; Katsumata *et al.*, 2022 ▸). The unit cell of Li_4_B_4_Al_3_O_12_Cl can be divided into eight equivalent cubic subcells, each containing one Cl1 at the cube centre. The Cl1 site is surrounded by six sites with multiplicity 24 (Wyckoff letter *c*) and four sites with multiplicity 32 (Wyckoff letter *e*) containing four Li atoms in total, and the resulting ClLi_4_ moiety is embedded in a negatively charged framework consisting of alternating corner-shared bridges of planar BO_3_ triangles and AlO_4_ tetra­hedra. The occupancy of Li1 is close to 1, whereas that of Li2 is ∼ 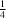 . The lattice parameter of Li_4_B_4_Al_3_O_12_Cl [*a* = 12.9839 (1) Å] is similar to that of the polycrystalline sample obtained by solid-state synthesis [*a* = 12.9687 (1) Å; Katsumata *et al.*, 2022 ▸] but larger than that crystallized from glass-ceramics [*a* = 12.9149 (5) Å; Kajihara *et al.*, 2017 ▸], probably because of an incomplete uptake of Al in crystals obtained from glass-ceramics.

Similar to Li_4_B_4_Al_3_O_12_Cl, the crystallites of Na_4_B_4_Al_3_O_12_Cl exhibit complete cross-polarized extinction. Refinement in the space group *F* 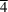 3*c* resulted in a reliability factor *R*1 of 0.022, and the results indicate that Na_4_B_4_Al_3_O_12_Cl is isostructural with Li_4_B_4_Al_3_O_12_Cl, except that Na1 is located at the 48 *g* site and displaced from the 24 *c* site at the midpoint between neighbouring Cl1 atoms. The lattice parameter of Na_4_B_4_Al_3_O_12_Cl [*a* = 13.5904 (1) Å] is the largest among known cubic boracites, apart from the sulfide variant Li_6_B_7_S_13_I [*a* = 15.245 (2) Å; Kaup *et al.*, 2021 ▸]. The occupancy of Cl1 is less than 1 (∼0.92), possibly because of its higher growth tem­per­ature compared to that of Li_4_B_4_Al_3_O_12_Cl. The equivalent isotropic displacement parameters (*U*_eq_) of extraframework species [Cl1, 0.0350 (9) Å^2^; Na1, 0.0422 (14) Å^2^; Na2, 0.017 (2) Å^2^] of Na_4_B_4_Al_3_O_12_Cl are notably smaller than those of Li_4_B_4_Al_3_O_12_Cl [Cl1, 0.0787 (15) Å^2^; Li1, 0.066 (6) Å^2^; Li2, 0.028 (11) Å^2^], despite that the species in the framework (B1, Al1 and O1) are similar or even larger in Na_4_B_4_Al_3_O_12_Cl. These observations suggest that replacing Li with Na increases the packing density at the extraframework sites and suppresses the thermal motion of the atoms located therein.

The convergence of the structure refinements of Li_4_B_4_Al_3_O_12_Cl and Na_4_B_4_Al_3_O_12_Cl in the space group *F* 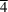 3*c* was satisfactory (*R*1 ≃ 0.03). However, these crystals both exhibit weak *hhl* reflections with odd *h* and *l*, which violate the extinction conditions in space group *F* 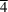 3*c*, as listed in Table 1 ▸. The noncentrosymmetric cubic space groups compatible with the observed reflection condition (*hhl*: *h* + *l* = 2*n*) are *F*23, *F*432 and *F* 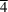 3*m*. Among them, only the structure analyses in the space group *F*23 were successful. The conversion of the space group from *F* 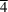 3*c* to *F*23 is accompanied by the splitting of atoms except for Al1. This conversion also splits Li1 at the 24 *c* site of *F* 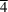 3*c* into Li1 and Li2 at the 24 *g* site of *F*23. The occupancies of *A*1 and *A*2 (*A* = Li, Na) converged to the upper bound [*g*(*A*1) = *g*(*A2*) = 0.5] both in Li_4_B_4_Al_3_O_12_Cl and Na_4_B_4_Al_3_O_12_Cl, and were fixed at this value. The slightly larger *R*1 factors in *F*23 compared to *F* 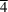 3*c* are due to an increased number of measured reflections partially with low intensities.

**Figure 1:**
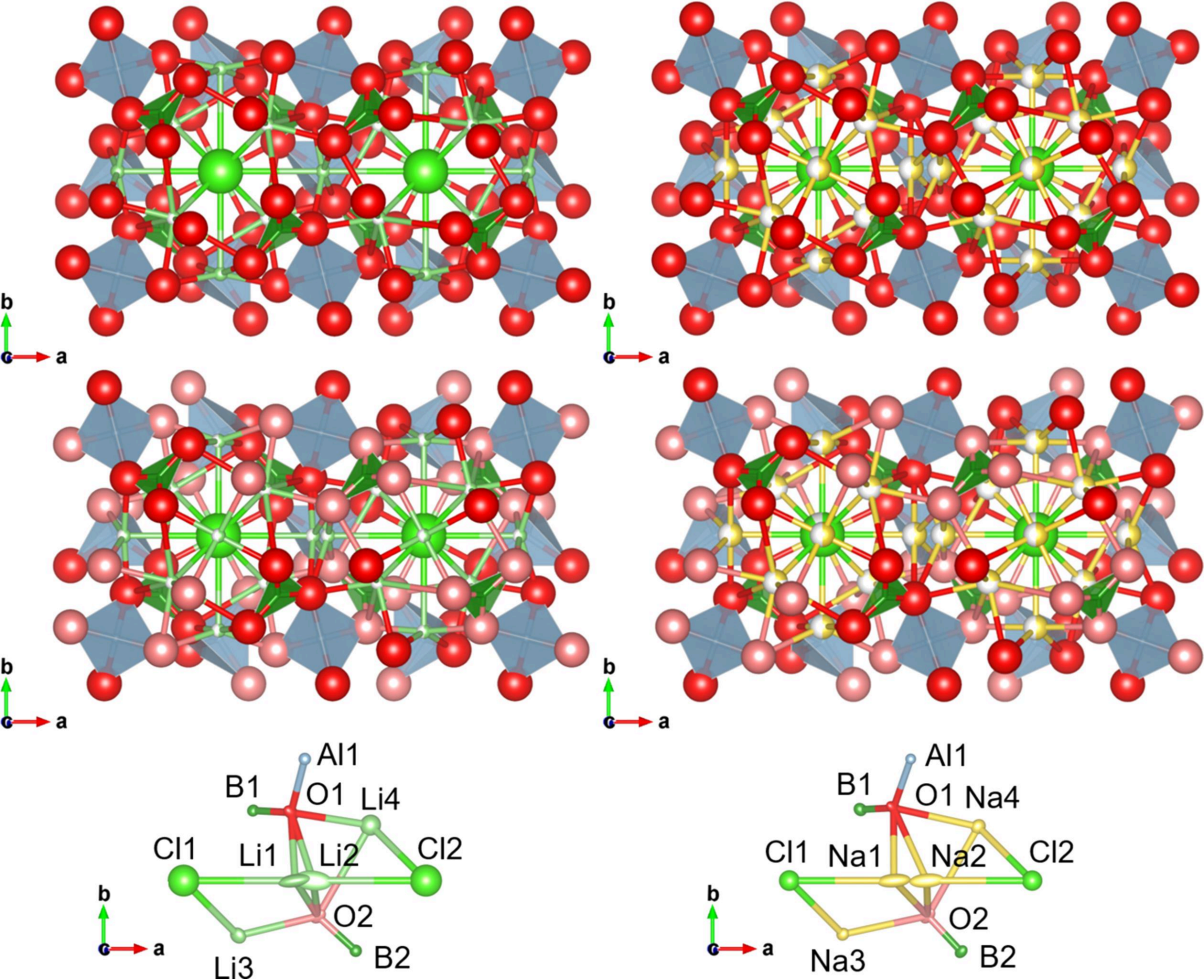
Schematic illustrations of two neighbouring cubic octant subcells in the unit cell of Li_4_B_4_Al_3_O_12_Cl in the space groups *F* 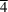 3*c* (top left) and *F*23 (middle left), those of Na_4_B_4_Al_3_O_12_Cl in the space groups *F* 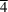 3*c* (top right) and *F*23 (middle right), and asymmetric units with displacement ellipsoids at the 50% probability level of Li_4_B_4_Al_3_O_12_Cl (bottom left) and Na_4_B_4_Al_3_O_12_Cl (bottom right) in the space group *F*23. Red, large green, small green and yellow spheres denote O, Cl, Li and Na atoms, respectively. Green triangles and gray tetra­hedra denote BO_3_ and AlO_4_ units, respectively. The forefront Al atoms located at *z* = 0.5 are not shown for clarity. In the middle and bottom figures, red and pale-red spheres denote O1 and O2 atoms, respectively.

Fig. 1 ▸ summarizes the schematic illustrations of two adjacent cubic octant subcells of the unit cells of Li_4_B_4_Al_3_O_12_Cl and Na_4_B_4_Al_3_O_12_Cl, along with their asymmetric units derived from the analyses in the space group *F*23. In the space group *F* 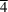 3*c*, the eight subcells in a unit cell are equivalent. In contrast, in the space group *F*23, they are classified into two types of subcells stacked alternately in three dimensions. Nevertheless, the atomic displacements associated with the conversion of the space group from *F* 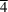 3*c* to *F*23 are small, and the structures solved in these two space groups are very similar, apart from the splitting of Li1 in space group *F* 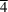 3*c* into Li1 and Li2 in space group *F*23. Such subcell ordering is also observed in the lithium-rich boracite Li_5_B_7_O_12.5_Cl (space group *F*23) (Vlasse *et al.*, 1981 ▸; Tezuka *et al.*, 2017 ▸; Li & Holzwarth, 2022 ▸); in Li_5_B_7_O_12.5_Cl, the chemical compositions of adjacent subcells differ notably as a result of the incorporation of excess Li and O and the resulting partial conversion of BO_3_ triangles to BO_4_ tetra­hedra, as well as the ordering of Li. In contrast, in the title compounds Li_4_B_4_Al_3_O_12_Cl and Na_4_B_4_Al_3_O_12_Cl, such a distinct structural ordering associated with a compositional change is not observed, as the conversion of BO_3_ triangles to BO_4_ tetra­hedra is unlikely even under alkali-rich conditions.

Table 2 ▸ lists selected atomic distances and angles. The B—O and Al—O distances are similar between Li_4_B_4_Al_3_O_12_Cl and Na_4_B_4_Al_3_O_12_Cl. In contrast, the B—O—Al angles in Na_4_B_4_Al_3_O_12_Cl are larger than those in Li_4_B_4_Al_3_O_12_Cl by ∼10°. This widening in the B—O—Al angles is responsible for the expansion of the unit cell in Na_4_B_4_Al_3_O_12_Cl. The increase in *A*—O (*A* = Li, Na) distances from Li_4_B_4_Al_3_O_12_Cl and Na_4_B_4_Al_3_O_12_Cl amounts to ∼0.2–0.3 Å, and is consistent with the difference in the ionic radii between Li and Na with the same coordination numbers (1.13–0.73 Å = 0.40 Å for fourfold corrdination and 1.16–0.90 Å = 0.26 Å for sixfold coordination; Shannon, 1976 ▸). In contrast, the increase in *A*—Cl distances is notably smaller (∼0.05 Å) or even negative, indicating an increase in the packing density of extraframework *A* and Cl. This observation is consistent with the smaller atomic displacement parameters of extraframework *A* and Cl in Na_4_B_4_Al_3_O_12_Cl compared to Li_4_B_4_Al_3_O_12_Cl (see above).

## 3. Synthesis and crystallization

Li_2_CO_3_ (Fujifilm Wako Chemicals, 99%), Na_2_CO_3_ (Fujifilm Wako Chemicals, 99%), B_2_O_3_ (Kojundo Chemical Laboratory, 99.9%), γ-Al_2_O_3_ (Kojundo Chemical Laboratory, 99.99%), LiCl (Kanto Chemical, 99.9%) and NaCl (Kojundo Chemical Laboratory, 99.9%) were mixed in an *A*_2_CO_3_:B_2_O_3_:γ-Al_2_O_3_:*A*Cl (*A* = Li, Na) molar ratio of 3:4:3:14, with a B_2_O_3_ content of 10 mmol. LiCl (melting point: 878 K) and NaCl (melting point: 1074 K), acting as self-fluxes, were added in excess. The mixture of sample 1 (*A* = Li) or 2 (*A* = Na) was placed in a platinum crucible covered by an alumina crucible, heated to 1073 K for 3 h (sample 1) or 1123 K for 4 h (sample 2), maintained for 5 h, cooled at a rate of 3 K h^−1^ for 50 h, and then cooled to room temperature in the furnace by turning off the power.

The resulting mixtures were washed with water to leach out water-soluble components. The residues were characterized by powder X-ray diffraction (SmartLab diffractometer, Rigaku) using Cu *K*α radiation. The main impurity phases in the residues of samples 1 and 2 were Li_2_BAlO_4_ (space group *P*2_1_/*c*) and LiAl_5_O_8_ (space group *P*4_1_32), and Na_2_Al_2_B_4_O_7_ (space group *P* 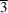 1*c*), respectively. Single-crystal particles with cubic symmetry were selected using an optical microscope (BH2, Olympus), showing complete light extinction under crossed polarizers.

## 4. Refinement

Crystal data, data collection and structure refinement details are summarized in Table 3 ▸. The crystal structures of Li_4_B_4_Al_3_O_12_Cl and Na_4_B_4_Al_3_O_12_Cl were solved both in the space groups *F* 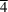 3*c* and *F*23 using the same *hkl* file. In the refinements in the space group *F*23, all reflections were used. In the refinements in the space group *F* 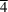 3*c*, reflections that violate the extinction conditions (885 in Li_4_B_4_Al_3_O_12_Cl and 1010 in Na_4_B_4_Al_3_O_12_Cl) were rejected by *SHELXL* (Sheldrick, 2015*b* ▸), but few rejected reflections had intensities with *I*/σ(*I*) > 3 (3 in Li_4_B_4_Al_3_O_12_Cl and 4 in Na_4_B_4_Al_3_O_12_Cl). To maintain charge neutrality, the occupancies, *g*, of *A* (*A* = Li, Na) and Cl were refined under the restraint that the total number of *A* in the unit cell was larger by 24 than that of Cl [*e.g.* 24*g*(Li1) + 32*g*(Li2) − 8*g*(Cl1) = 24 for Li_4_B_4_Al_3_O_12_Cl solved in *F* 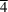 3*c*], while permitting possible Cl deficiency. The summary of reflections was derived by the program *SpaceGroup* in *WinGX* (Farrugia, 2012 ▸). 

## Supplementary Material

Crystal structure: contains datablock(s) kjh0818yoshinoL14_0m_a, kjh0818yoshinoL14_0m_a_1, kjh230804yoshinoN4_0m_a, kjh230804yoshinoN4_0m_a_1, global. DOI: 10.1107/S2056989024000501/wm5704sup1.cif

Structure factors: contains datablock(s) kjh0818yoshinoL14_0m_a. DOI: 10.1107/S2056989024000501/wm5704kjh0818yoshinoL14_0m_asup2.hkl

Structure factors: contains datablock(s) kjh0818yoshinoL14_0m_a_1. DOI: 10.1107/S2056989024000501/wm5704kjh0818yoshinoL14_0m_a_1sup3.hkl

Structure factors: contains datablock(s) kjh230804yoshinoN4_0m_a. DOI: 10.1107/S2056989024000501/wm5704kjh230804yoshinoN4_0m_asup4.hkl

Structure factors: contains datablock(s) kjh230804yoshinoN4_0m_a_1. DOI: 10.1107/S2056989024000501/wm5704kjh230804yoshinoN4_0m_a_1sup5.hkl

CCDC references: 2325537, 2325536, 2325535, 2325534

Additional supporting information:  crystallographic information; 3D view; checkCIF report

## Figures and Tables

**Table 1:** Summary of the observed *hhl* reflections in Li_4_B_4_Al_3_O_12_Cl and Na_4_B_4_Al_3_O_12_Cl

	Li_4_B_4_Al_3_O_12_Cl		Na_4_B_4_Al_3_O_12_Cl	
	<*I*/σ(*I*)>	Number of reflections	<*I*/σ(*I*)>	Number of reflections
No conditions	14.35	325	12.14	340
*h* even	30.24	153	24.96	165
*h* odd	0.21	172	0.04	175
*l* even	30.24	153	24.96	165
*l* odd	0.21	172	0.04	175
*h* + *l* even	14.35	325	12.14	340
*h* + *l* odd	0.00	0	0.00	0

**Table 2:** Selected bond lengths and angles (Å, °) in *A*_4_B_4_Al_3_O_12_Cl crystal structures

Space group *F* 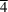 3*c*			Space group *F*23		
	*A* = Li	*A* = Na		*A* = Li	*A* = Na
*A*1—O1	2.0828 (17)	2.2588 (16)	*A*1—O1	2.053 (3)	2.2585 (18)
*A*1—O1^i^	2.0828 (17)	2.446 (2)	*A*1—O2^ii^	2.143 (16)	2.452 (3)
			*A*2—O2^iii^	2.056 (4)	2.2602 (18)
			*A*2—O1^iv^	2.132 (16)	2.442 (3)
*A*1—Cl1	3.2460 (1)	2.936 (4)	*A*1—Cl1	2.98 (6)	2.925 (6)
			*A*2—Cl2	3.03 (6)	2.948 (6)
*A*2—O1^v^	2.22 (3)	2.484 (5)	*A*3—O2^vi^	2.23 (3)	2.488 (6)
*A*2—O1^vii^	2.828 (8)	2.913 (2)	*A*3—O1^vii^	2.824 (8)	2.912 (2)
			*A*4—O1^viii^	2.23 (3)	2.480 (6)
			*A*4—O2^ix^	2.826 (8)	2.914 (2)
*A*2—Cl1	2.57 (5)	2.605 (9)	*A*3—Cl1	2.55 (6)	2.599 (10)
			*A*4—Cl2	2.55 (5)	2.612 (10)
B1—O1	1.3700 (16)	1.3693 (15)	B1—O1	1.3693 (17)	1.3684 (16)
			B2—O2^x^	1.3702 (17)	1.3684 (16)
Al1—O1^xi^	1.7533 (17)	1.7506 (16)	Al1—O1^xi^	1.754 (2)	1.7495 (18)
			Al1—O2^xii^	1.754 (2)	1.7522 (18)
					
B1—O1—Al1^xiii^	118.97 (12)	128.59 (12)	B1—O1—Al1^xiii^	119.05 (13)	128.62 (14)
			B2^xiv^—O2—Al1^vii^	118.94 (13)	128.66 (14)

Symmetry code(*s*): (i) −*x*, −*z* + 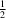 , *y*; (ii) −*x*, *y*, −*z* + 1; (iii) *x* + 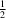 , −*y* + 1, −*z* + 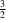 ; (iv) −*x* + 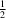 , −*y* + 1, *z* + 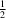 ; (v) −*y* + 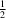 , *x* + 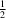 , −*z* + 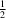 ; (vi) −*z* + 1, *x* + 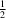 , −*y* + 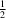 ; (vii) *z*, −*x* + 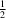 , −*y* + 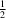 ; (viii) *x* + 1, −*y* + 1, −*z* + 1; (ix) −*x* + 1, *y* + 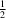 , −*z* + 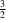 ; (x) *z*, *x* + 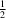 , *y* + 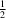 ; (xi) *z*, *x*, *y*; (xii) −*y* + 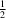 , *z* − 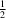 , −*x*; (xiii) *y*, *z*, *x*; (xiv) *x* − 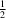 , *y* − 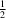 , *z*.

**Table 3:** Experimental details

	**Li_4_B_4_Al_3_O_12_Cl in *F* 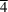 3*c***	**Li_4_B_4_Al_3_O_12_Cl in *F*23**	**Na_3.92_B_4_Al_3_O_12_Cl_0.92_ in *F* 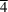 3*c***	**Na_3.92_B_4_Al_3_O_12_Cl_0.92_ in *F*23**
Crystal data				
Chemical formula	Li_4_B_4_Al_3_O_12_Cl	Li_4_B_4_Al_3_O_12_Cl	Na_3.92_B_4_Al_3_O_12_Cl_0.92_	Na_3.92_B_4_Al_3_O_12_Cl_0.92_
*M*_r_	379.39	379.39	438.91	438.91
Crystal system, space group	Cubic, *F* 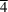 3*c*	Cubic, *F*23	Cubic, *F* 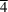 3*c*	Cubic, *F*23
Temperature (K)	297	297	294	294
*a* (Å)	12.9839 (1)	12.9839 (1)	13.5904 (1)	13.5904 (1)
*V* (Å^3^)	2188.85 (5)	2188.85 (5)	2510.13 (6)	2510.13 (6)
*Z*	8	8	8	8
Radiation type	Cu *K*α	Cu *K*α	Cu *K*α	Cu *K*α
μ (mm^−1^)	6.12	6.12	6.78	6.78
Crystal size (mm)	0.11 × 0.10 × 0.06	0.11 × 0.10 × 0.06	0.08 × 0.06 × 0.04	0.08 × 0.06 × 0.04

Data collection				
Diffractometer	Bruker D8 goniometer	Bruker D8 goniometer	Bruker D8 goniometer	Bruker D8 goniometer
Absorption correction	Multi-scan (*SADABS*; Krause *et al.*, 2015 ▸)	Multi-scan (*SADABS*; Krause *et al.*, 2015 ▸)	Multi-scan (*SADABS*; Krause *et al.*, 2015 ▸)	Multi-scan (*SADABS*; Krause *et al.*, 2015 ▸)
*T*_min_, *T*_max_	0.62, 0.75	0.62, 0.75	0.66, 0.75	0.66, 0.75
No. of measured, independent and observed [*I* > 2σ(*I*)] reflections	3843, 193, 191	4728, 389, 317	4504, 218, 216	5514, 437, 355
*R*_int_	0.023	0.024	0.027	0.028
(sin θ/λ)_max_ (Å^−1^)	0.621	0.621	0.624	0.624

Refinement				
*R*[*F* ^2^ > 2σ(*F* ^2^)], *wR*(*F* ^2^), *S*	0.031, 0.075, 1.16	0.032, 0.081, 1.17	0.022, 0.055, 1.20	0.024, 0.066, 1.12
No. of reflections	193	389	218	437
No. of parameters	23	50	27	52
No. of restraints	1	1	1	1
Δρ_max_, Δρ_min_ (e Å^−3^)	0.20, −0.63	0.20, −0.91	0.15, −0.45	0.17, −0.58
Absolute structure	Flack *x* determined using 80 quotients [(*I* ^+^) − (*I* ^−^)]/[(*I* ^+^) + (*I* ^−^)] (Parsons *et al.*, 2013 ▸)	Flack *x* determined using 132 quotients [(*I* ^+^) − (*I* ^−^)]/[(*I* ^+^) + (*I* ^−^)] (Parsons *et al.*, 2013 ▸).	Flack *x* determined using 89 quotients [(*I* ^+^) − (*I* ^−^)]/[(*I* ^+^) + (*I* ^−^)] (Parsons *et al.*, 2013 ▸)	Flack *x* determined using 142 quotients [(*I* ^+^) − (*I* ^−^)]/[(*I* ^+^) + (*I* ^−^)] (Parsons *et al.*, 2013 ▸).
Absolute structure parameter	−0.03 (3)	−0.03 (2)	0.01 (3)	0.01 (2)

Computer programs: *BIS* (Bruker, 2021 ▸), *SAINT* (Bruker, 2019 ▸), *SHELXTL* (Sheldrick, 2015*a* ▸), *SHELXL* (Sheldrick, 2015*b* ▸), *ShelXle* (Hübschle *et al.*, 2011 ▸), *VESTA* (Momma & Izumi, 2011 ▸) and *publCIF* (Westrip, 2010 ▸).

## supplementary crystallographic information

### Lithium aluminoboracite (kjh0818yoshinoL14_0m_a) Crystal data

**Table:**

Li_4_B_4_Al_3_O_12_Cl	Cu *K*α radiation, λ = 1.54178 Å
*M**_r_* = 379.39	Cell parameters from 2808 reflections
Cubic, *F*43*c*	θ = 6.8–73.2°
*a* = 12.9839 (1) Å	µ = 6.12 mm^−^^1^
*V* = 2188.85 (5) Å^3^	*T* = 297 K
*Z* = 8	Plate, colorless
*F*(000) = 1472	0.11 × 0.10 × 0.06 mm
*D*_x_ = 2.303 Mg m^−^^3^	

### Lithium aluminoboracite (kjh0818yoshinoL14_0m_a) Data collection

**Table:**

Bruker D8 goniometer diffractometer	193 independent reflections
Radiation source: sealed tube	191 reflections with *I* > 2σ(*I*)
Detector resolution: 7.3910 pixels mm^-1^	*R*_int_ = 0.023
ω scans	θ_max_ = 73.2°, θ_min_ = 6.8°
Absorption correction: multi-scan (*SADABS*; Krause *et al.*, 2015)	*h* = −15→12
*T*_min_ = 0.62, *T*_max_ = 0.75	*k* = −15→16
3843 measured reflections	*l* = −16→15

### Lithium aluminoboracite (kjh0818yoshinoL14_0m_a) Refinement

**Table:**

Refinement on *F*^2^	1 restraint
Least-squares matrix: full	*w* = 1/[σ^2^(*F*_o_^2^) + (0.0536*P*)^2^ + 3.2978*P*] where *P* = (*F*_o_^2^ + 2*F*_c_^2^)/3
*R*[*F*^2^ > 2σ(*F*^2^)] = 0.031	(Δ/σ)_max_ < 0.001
*wR*(*F*^2^) = 0.075	Δρ_max_ = 0.20 e Å^−^^3^
*S* = 1.16	Δρ_min_ = −0.63 e Å^−^^3^
193 reflections	Absolute structure: Flack *x* determined using 80 quotients [(I+)-(I-)]/[(I+)+(I-)] (Parsons *et al.*, 2013)
23 parameters	Absolute structure parameter: −0.03 (3)

### Lithium aluminoboracite (kjh0818yoshinoL14_0m_a) Special details

**Table:**

Geometry. All esds (except the esd in the dihedral angle between two l.s. planes) are estimated using the full covariance matrix. The cell esds are taken into account individually in the estimation of esds in distances, angles and torsion angles; correlations between esds in cell parameters are only used when they are defined by crystal symmetry. An approximate (isotropic) treatment of cell esds is used for estimating esds involving l.s. planes.

### Lithium aluminoboracite (kjh0818yoshinoL14_0m_a) Fractional atomic coordinates and isotropic or equivalent isotropic displacement parameters (Å^2^)

**Table:**

	*x*	*y*	*z*	*U*_iso_*/*U*_eq_	Occ. (<1)
Li1	0.000000	0.250000	0.250000	0.066 (6)	0.991 (13)
Li2	0.364 (2)	0.364 (2)	0.364 (2)	0.028 (11)	0.256 (10)
B1	0.1057 (3)	0.1057 (3)	0.1057 (3)	0.0114 (10)	
Al1	0.250000	0.000000	0.000000	0.0118 (6)	
O1	0.02786 (13)	0.11000 (13)	0.17680 (15)	0.0166 (6)	
Cl1	0.250000	0.250000	0.250000	0.0787 (15)	

### Lithium aluminoboracite (kjh0818yoshinoL14_0m_a) Atomic displacement parameters (Å^2^)

**Table:**

	*U*^11^	*U*^22^	*U*^33^	*U*^12^	*U*^13^	*U*^23^
Li1	0.156 (18)	0.021 (4)	0.021 (4)	0.000	0.000	0.000
Li2	0.028 (11)	0.028 (11)	0.028 (11)	0.000 (9)	0.000 (9)	0.000 (9)
B1	0.0114 (10)	0.0114 (10)	0.0114 (10)	−0.0014 (14)	−0.0014 (14)	−0.0014 (14)
Al1	0.0115 (8)	0.0120 (6)	0.0120 (6)	0.000	0.000	0.000
O1	0.0185 (11)	0.0154 (10)	0.0158 (9)	0.0048 (7)	0.0067 (10)	0.0044 (9)
Cl1	0.0787 (15)	0.0787 (15)	0.0787 (15)	0.000	0.000	0.000

### Lithium aluminoboracite (kjh0818yoshinoL14_0m_a) Geometric parameters (Å, º)

**Table:**

Li1—O1	2.0828 (17)	Li2—O1^xiii^	2.22 (3)
Li1—O1^i^	2.0828 (17)	Li2—Cl1	2.57 (5)
Li1—O1^ii^	2.0828 (17)	Li2—O1^ix^	2.828 (8)
Li1—O1^iii^	2.0828 (17)	Li2—O1^xiv^	2.828 (8)
Li1—Li2^iv^	2.740 (13)	Li2—O1^vi^	2.828 (8)
Li1—Li2^v^	2.740 (13)	Li2—Al1^xv^	2.90 (2)
Li1—Li2^vi^	2.740 (13)	Li2—Al1^xvi^	2.90 (2)
Li1—Li2^vii^	2.740 (13)	B1—O1^x^	1.3700 (16)
Li1—Cl1	3.2460 (1)	B1—O1^xvii^	1.3700 (16)
Li1—Al1^viii^	3.2460 (1)	B1—O1	1.3700 (16)
Li1—Al1^ix^	3.2460 (1)	Al1—O1^xviii^	1.7533 (17)
Li1—Al1^x^	3.2460 (1)	Al1—O1^xix^	1.7533 (17)
Li2—O1^xi^	2.22 (3)	Al1—O1^xvii^	1.7533 (17)
Li2—O1^xii^	2.22 (3)	Al1—O1^xx^	1.7533 (17)
			
O1—Li1—O1^i^	160.00 (10)	O1^ix^—Li2—O1^vi^	118.0 (5)
O1—Li1—O1^ii^	91.729 (17)	O1^xiv^—Li2—O1^vi^	118.0 (5)
O1^i^—Li1—O1^ii^	91.729 (17)	O1^xi^—Li2—Al1^xv^	69.0 (7)
O1—Li1—O1^iii^	91.729 (17)	O1^xii^—Li2—Al1^xv^	37.1 (3)
O1^i^—Li1—O1^iii^	91.729 (17)	O1^xiii^—Li2—Al1^xv^	123 (2)
O1^ii^—Li1—O1^iii^	160.00 (10)	Cl1—Li2—Al1^xv^	114.0 (9)
O1—Li1—Li2^iv^	52.6 (10)	Li1^xiv^—Li2—Al1^xv^	171 (2)
O1^i^—Li1—Li2^iv^	146.2 (9)	Li1^ix^—Li2—Al1^xv^	70.20 (14)
O1^ii^—Li1—Li2^iv^	70.29 (8)	Li1^vi^—Li2—Al1^xv^	70.20 (14)
O1^iii^—Li1—Li2^iv^	96.5 (4)	O1^ix^—Li2—Al1^xv^	106.4 (4)
O1—Li1—Li2^v^	96.5 (4)	O1^xiv^—Li2—Al1^xv^	134.8 (7)
O1^i^—Li1—Li2^v^	70.29 (8)	O1^vi^—Li2—Al1^xv^	35.62 (12)
O1^ii^—Li1—Li2^v^	52.6 (10)	O1^xi^—Li2—Al1^xvi^	123 (2)
O1^iii^—Li1—Li2^v^	146.2 (9)	O1^xii^—Li2—Al1^xvi^	69.0 (7)
Li2^iv^—Li1—Li2^v^	114.4 (12)	O1^xiii^—Li2—Al1^xvi^	37.1 (3)
O1—Li1—Li2^vi^	70.29 (8)	Cl1—Li2—Al1^xvi^	114.0 (9)
O1^i^—Li1—Li2^vi^	96.5 (4)	Li1^xiv^—Li2—Al1^xvi^	70.20 (14)
O1^ii^—Li1—Li2^vi^	146.2 (9)	Li1^ix^—Li2—Al1^xvi^	70.20 (14)
O1^iii^—Li1—Li2^vi^	52.6 (10)	Li1^vi^—Li2—Al1^xvi^	171 (2)
Li2^iv^—Li1—Li2^vi^	114.4 (12)	O1^ix^—Li2—Al1^xvi^	35.62 (12)
Li2^v^—Li1—Li2^vi^	100 (2)	O1^xiv^—Li2—Al1^xvi^	106.4 (4)
O1—Li1—Li2^vii^	146.2 (9)	O1^vi^—Li2—Al1^xvi^	134.8 (7)
O1^i^—Li1—Li2^vii^	52.6 (10)	Al1^xv^—Li2—Al1^xvi^	104.6 (11)
O1^ii^—Li1—Li2^vii^	96.5 (4)	O1^x^—B1—O1^xvii^	119.981 (12)
O1^iii^—Li1—Li2^vii^	70.29 (8)	O1^x^—B1—O1	119.980 (12)
Li2^iv^—Li1—Li2^vii^	100 (2)	O1^xvii^—B1—O1	119.982 (12)
Li2^v^—Li1—Li2^vii^	114.4 (12)	O1^xviii^—Al1—O1^xix^	107.09 (6)
Li2^vi^—Li1—Li2^vii^	114.4 (12)	O1^xviii^—Al1—O1^xvii^	114.35 (13)
O1—Li1—Cl1	80.00 (5)	O1^xix^—Al1—O1^xvii^	107.09 (6)
O1^i^—Li1—Cl1	80.00 (5)	O1^xviii^—Al1—O1^xx^	107.09 (6)
O1^ii^—Li1—Cl1	100.00 (5)	O1^xix^—Al1—O1^xx^	114.35 (13)
O1^iii^—Li1—Cl1	100.00 (5)	O1^xvii^—Al1—O1^xx^	107.09 (6)
Li2^iv^—Li1—Cl1	130.0 (11)	O1^xviii^—Al1—Li2^i^	153.8 (2)
Li2^v^—Li1—Cl1	50.0 (11)	O1^xix^—Al1—Li2^i^	49.7 (3)
Li2^vi^—Li1—Cl1	50.0 (11)	O1^xvii^—Al1—Li2^i^	69.9 (9)
Li2^vii^—Li1—Cl1	130.0 (11)	O1^xx^—Al1—Li2^i^	95.3 (7)
O1—Li1—Al1^viii^	150.78 (5)	O1^xviii^—Al1—Li2^xxi^	69.9 (9)
O1^i^—Li1—Al1^viii^	29.22 (5)	O1^xix^—Al1—Li2^xxi^	95.3 (7)
O1^ii^—Li1—Al1^viii^	62.85 (5)	O1^xvii^—Al1—Li2^xxi^	153.8 (2)
O1^iii^—Li1—Al1^viii^	117.15 (5)	O1^xx^—Al1—Li2^xxi^	49.7 (3)
Li2^iv^—Li1—Al1^viii^	122.8 (6)	Li2^i^—Al1—Li2^xxi^	118.5 (19)
Li2^v^—Li1—Al1^viii^	57.2 (6)	O1^xviii^—Al1—Li2^xxii^	95.3 (7)
Li2^vi^—Li1—Al1^viii^	122.8 (6)	O1^xix^—Al1—Li2^xxii^	153.8 (2)
Li2^vii^—Li1—Al1^viii^	57.2 (6)	O1^xvii^—Al1—Li2^xxii^	49.7 (3)
Cl1—Li1—Al1^viii^	90.0	O1^xx^—Al1—Li2^xxii^	69.9 (9)
O1—Li1—Al1^ix^	117.15 (5)	Li2^i^—Al1—Li2^xxii^	105.2 (9)
O1^i^—Li1—Al1^ix^	62.85 (5)	Li2^xxi^—Al1—Li2^xxii^	105.2 (9)
O1^ii^—Li1—Al1^ix^	150.78 (5)	O1^xviii^—Al1—Li2^xxiii^	49.7 (3)
O1^iii^—Li1—Al1^ix^	29.22 (5)	O1^xix^—Al1—Li2^xxiii^	69.9 (9)
Li2^iv^—Li1—Al1^ix^	122.8 (6)	O1^xvii^—Al1—Li2^xxiii^	95.3 (7)
Li2^v^—Li1—Al1^ix^	122.8 (6)	O1^xx^—Al1—Li2^xxiii^	153.8 (2)
Li2^vi^—Li1—Al1^ix^	57.2 (6)	Li2^i^—Al1—Li2^xxiii^	105.2 (9)
Li2^vii^—Li1—Al1^ix^	57.2 (6)	Li2^xxi^—Al1—Li2^xxiii^	105.2 (9)
Cl1—Li1—Al1^ix^	90.0	Li2^xxii^—Al1—Li2^xxiii^	118.5 (19)
Al1^viii^—Li1—Al1^ix^	90.0	O1^xviii^—Al1—Li1^xxiv^	78.09 (5)
O1—Li1—Al1^x^	29.22 (5)	O1^xix^—Al1—Li1^xxiv^	144.55 (6)
O1^i^—Li1—Al1^x^	150.78 (5)	O1^xvii^—Al1—Li1^xxiv^	101.91 (5)
O1^ii^—Li1—Al1^x^	117.15 (5)	O1^xx^—Al1—Li1^xxiv^	35.45 (6)
O1^iii^—Li1—Al1^x^	62.85 (5)	Li2^i^—Al1—Li1^xxiv^	127.4 (4)
Li2^iv^—Li1—Al1^x^	57.2 (6)	Li2^xxi^—Al1—Li1^xxiv^	52.6 (4)
Li2^v^—Li1—Al1^x^	122.8 (6)	Li2^xxii^—Al1—Li1^xxiv^	52.6 (4)
Li2^vi^—Li1—Al1^x^	57.2 (6)	Li2^xxiii^—Al1—Li1^xxiv^	127.4 (4)
Li2^vii^—Li1—Al1^x^	122.8 (6)	O1^xviii^—Al1—Li1^x^	101.91 (5)
Cl1—Li1—Al1^x^	90.0	O1^xix^—Al1—Li1^x^	35.45 (6)
Al1^viii^—Li1—Al1^x^	180.0	O1^xvii^—Al1—Li1^x^	78.09 (5)
Al1^ix^—Li1—Al1^x^	90.0	O1^xx^—Al1—Li1^x^	144.55 (6)
O1^xi^—Li2—O1^xii^	97.0 (15)	Li2^i^—Al1—Li1^x^	52.6 (4)
O1^xi^—Li2—O1^xiii^	97.0 (15)	Li2^xxi^—Al1—Li1^x^	127.4 (4)
O1^xii^—Li2—O1^xiii^	97.0 (15)	Li2^xxii^—Al1—Li1^x^	127.4 (4)
O1^xi^—Li2—Cl1	120.1 (12)	Li2^xxiii^—Al1—Li1^x^	52.6 (4)
O1^xii^—Li2—Cl1	120.1 (12)	Li1^xxiv^—Al1—Li1^x^	180.0
O1^xiii^—Li2—Cl1	120.1 (12)	O1^xviii^—Al1—Li1^xvii^	144.55 (6)
O1^xi^—Li2—Li1^xiv^	107.0 (3)	O1^xix^—Al1—Li1^xvii^	101.91 (5)
O1^xii^—Li2—Li1^xiv^	139.1 (8)	O1^xvii^—Al1—Li1^xvii^	35.45 (6)
O1^xiii^—Li2—Li1^xiv^	48.29 (9)	O1^xx^—Al1—Li1^xvii^	78.09 (5)
Cl1—Li2—Li1^xiv^	75.3 (11)	Li2^i^—Al1—Li1^xvii^	52.6 (4)
O1^xi^—Li2—Li1^ix^	139.1 (8)	Li2^xxi^—Al1—Li1^xvii^	127.4 (4)
O1^xii^—Li2—Li1^ix^	48.29 (9)	Li2^xxii^—Al1—Li1^xvii^	52.6 (4)
O1^xiii^—Li2—Li1^ix^	107.0 (3)	Li2^xxiii^—Al1—Li1^xvii^	127.4 (4)
Cl1—Li2—Li1^ix^	75.3 (11)	Li1^xxiv^—Al1—Li1^xvii^	90.0
Li1^xiv^—Li2—Li1^ix^	113.8 (9)	Li1^x^—Al1—Li1^xvii^	90.0
O1^xi^—Li2—Li1^vi^	48.29 (9)	O1^xviii^—Al1—Li1^xviii^	35.45 (6)
O1^xii^—Li2—Li1^vi^	107.0 (3)	O1^xix^—Al1—Li1^xviii^	78.09 (5)
O1^xiii^—Li2—Li1^vi^	139.1 (8)	O1^xvii^—Al1—Li1^xviii^	144.55 (6)
Cl1—Li2—Li1^vi^	75.3 (11)	O1^xx^—Al1—Li1^xviii^	101.91 (5)
Li1^xiv^—Li2—Li1^vi^	113.8 (9)	Li2^i^—Al1—Li1^xviii^	127.4 (4)
Li1^ix^—Li2—Li1^vi^	113.8 (9)	Li2^xxi^—Al1—Li1^xviii^	52.6 (4)
O1^xi^—Li2—O1^ix^	158 (2)	Li2^xxii^—Al1—Li1^xviii^	127.4 (4)
O1^xii^—Li2—O1^ix^	71.54 (19)	Li2^xxiii^—Al1—Li1^xviii^	52.6 (4)
O1^xiii^—Li2—O1^ix^	66.73 (16)	Li1^xxiv^—Al1—Li1^xviii^	90.0
Cl1—Li2—O1^ix^	81.8 (11)	Li1^x^—Al1—Li1^xviii^	90.0
Li1^xiv^—Li2—O1^ix^	74.1 (3)	Li1^xvii^—Al1—Li1^xviii^	180.0
Li1^ix^—Li2—O1^ix^	43.90 (17)	B1—O1—Al1^x^	118.97 (12)
Li1^vi^—Li2—O1^ix^	152.3 (17)	B1—O1—Li1	118.1 (2)
O1^xi^—Li2—O1^xiv^	66.73 (16)	Al1^x^—O1—Li1	115.33 (9)
O1^xii^—Li2—O1^xiv^	158 (2)	B1—O1—Li2^iv^	123.5 (10)
O1^xiii^—Li2—O1^xiv^	71.54 (19)	Al1^x^—O1—Li2^iv^	93.18 (8)
Cl1—Li2—O1^xiv^	81.8 (11)	Li1—O1—Li2^iv^	79.2 (11)
Li1^xiv^—Li2—O1^xiv^	43.90 (17)	Li2—Cl1—Li2^i^	109.471 (6)
Li1^ix^—Li2—O1^xiv^	152.3 (17)	Li2—Cl1—Li2^vi^	109.471 (1)
Li1^vi^—Li2—O1^xiv^	74.1 (3)	Li2^i^—Cl1—Li2^vi^	109.5
O1^ix^—Li2—O1^xiv^	118.0 (5)	Li2—Cl1—Li2^v^	109.471 (3)
O1^xi^—Li2—O1^vi^	71.54 (19)	Li2^i^—Cl1—Li2^v^	109.471 (1)
O1^xii^—Li2—O1^vi^	66.73 (16)	Li2^vi^—Cl1—Li2^v^	109.471 (1)
O1^xiii^—Li2—O1^vi^	158 (2)	Li2—Cl1—Li1	125.264 (1)
Cl1—Li2—O1^vi^	81.8 (11)	Li2^i^—Cl1—Li1	125.264 (1)
Li1^xiv^—Li2—O1^vi^	152.3 (17)	Li2^vi^—Cl1—Li1	54.736 (1)
Li1^ix^—Li2—O1^vi^	74.1 (3)	Li2^v^—Cl1—Li1	54.736 (1)
Li1^vi^—Li2—O1^vi^	43.90 (17)		

Symmetry codes: (i) *x*, −*y*+1/2, −*z*+1/2; (ii) −*x*, −*z*+1/2, *y*; (iii) −*x*, *z*, −*y*+1/2; (iv) *y*−1/2, −*x*+1/2, −*z*+1/2; (v) −*x*+1/2, *y*, −*z*+1/2; (vi) −*x*+1/2, −*y*+1/2, *z*; (vii) *y*−1/2, *x*, *z*; (viii) −*y*, *z*+1/2, −*x*+1/2; (ix) *z*, −*x*+1/2, −*y*+1/2; (x) *y*, *z*, *x*; (xi) *x*+1/2, −*z*+1/2, −*y*+1/2; (xii) −*y*+1/2, *x*+1/2, −*z*+1/2; (xiii) −*z*+1/2, −*y*+1/2, *x*+1/2; (xiv) −*y*+1/2, *z*, −*x*+1/2; (xv) *y*+1/2, *z*+1/2, *x*; (xvi) *x*, *y*+1/2, *z*+1/2; (xvii) *z*, *x*, *y*; (xviii) *z*, −*x*, −*y*; (xix) −*z*+1/2, *y*, −*x*; (xx) −*z*+1/2, −*y*, *x*; (xxi) *x*, *y*−1/2, *z*−1/2; (xxii) −*y*+1/2, *x*−1/2, −*z*+1/2; (xxiii) −*y*+1/2, −*x*+1/2, *z*−1/2; (xxiv) −*y*+1/2, *z*−1/2, −*x*.

### Lithium aluminoboracite (kjh0818yoshinoL14_0m_a_1) Crystal data

**Table:**

Li_4_B_4_Al_3_O_12_Cl	Cu *K*α radiation, λ = 1.54178 Å
*M**_r_* = 379.39	Cell parameters from 2808 reflections
Cubic, *F*23	θ = 6.8–73.2°
*a* = 12.9839 (1) Å	µ = 6.12 mm^−^^1^
*V* = 2188.85 (5) Å^3^	*T* = 297 K
*Z* = 8	Plate, colorless
*F*(000) = 1472	0.11 × 0.10 × 0.06 mm
*D*_x_ = 2.303 Mg m^−^^3^	

### Lithium aluminoboracite (kjh0818yoshinoL14_0m_a_1) Data collection

**Table:**

Bruker D8 goniometer diffractometer	389 independent reflections
Radiation source: sealed tube	317 reflections with *I* > 2σ(*I*)
Detector resolution: 7.3910 pixels mm^-1^	*R*_int_ = 0.024
ω scans	θ_max_ = 73.2°, θ_min_ = 5.9°
Absorption correction: multi-scan (*SADABS*; Krause *et al.*, 2015)	*h* = −15→12
*T*_min_ = 0.62, *T*_max_ = 0.75	*k* = −15→16
4728 measured reflections	*l* = −16→15

### Lithium aluminoboracite (kjh0818yoshinoL14_0m_a_1) Refinement

**Table:**

Refinement on *F*^2^	1 restraint
Least-squares matrix: full	*w* = 1/[σ^2^(*F*_o_^2^) + (0.0537*P*)^2^ + 1.5774*P*] where *P* = (*F*_o_^2^ + 2*F*_c_^2^)/3
*R*[*F*^2^ > 2σ(*F*^2^)] = 0.032	(Δ/σ)_max_ = 0.003
*wR*(*F*^2^) = 0.081	Δρ_max_ = 0.20 e Å^−^^3^
*S* = 1.17	Δρ_min_ = −0.91 e Å^−^^3^
389 reflections	Absolute structure: Flack *x* determined using 132 quotients [(I+)-(I-)]/[(I+)+(I-)] (Parsons *et al.*, 2013).
50 parameters	Absolute structure parameter: −0.03 (2)

### Lithium aluminoboracite (kjh0818yoshinoL14_0m_a_1) Special details

**Table:**

Geometry. All esds (except the esd in the dihedral angle between two l.s. planes) are estimated using the full covariance matrix. The cell esds are taken into account individually in the estimation of esds in distances, angles and torsion angles; correlations between esds in cell parameters are only used when they are defined by crystal symmetry. An approximate (isotropic) treatment of cell esds is used for estimating esds involving l.s. planes.

### Lithium aluminoboracite (kjh0818yoshinoL14_0m_a_1) Fractional atomic coordinates and isotropic or equivalent isotropic displacement parameters (Å^2^)

**Table:**

	*x*	*y*	*z*	*U*_iso_*/*U*_eq_	Occ. (<1)
Li1	0.020 (4)	0.250000	0.250000	0.047 (13)	0.5
Li2	0.517 (5)	0.750000	0.750000	0.050 (15)	0.5
Li3	0.363 (3)	0.363 (3)	0.363 (3)	0.020 (12)	0.205 (14)
Li4	0.864 (2)	0.864 (2)	0.864 (2)	0.035 (12)	0.295 (14)
B1	0.1060 (3)	0.1060 (3)	0.1060 (3)	0.0112 (10)	
B2	0.6057 (3)	0.6057 (3)	0.6057 (3)	0.0119 (10)	
Al1	0.24997 (11)	0.000000	0.000000	0.0118 (5)	
O1	0.02797 (13)	0.11005 (14)	0.17683 (14)	0.0170 (5)	
O2	0.02784 (13)	0.17681 (14)	0.61004 (14)	0.0171 (5)	
Cl1	0.250000	0.250000	0.250000	0.0774 (16)	
Cl2	0.750000	0.750000	0.750000	0.0786 (16)	

### Lithium aluminoboracite (kjh0818yoshinoL14_0m_a_1) Atomic displacement parameters (Å^2^)

**Table:**

	*U*^11^	*U*^22^	*U*^33^	*U*^12^	*U*^13^	*U*^23^
Li1	0.08 (3)	0.015 (11)	0.040 (17)	0.000	0.000	−0.012 (14)
Li2	0.11 (5)	0.031 (14)	0.010 (11)	0.000	0.000	−0.008 (13)
Li3	0.020 (12)	0.020 (12)	0.020 (12)	−0.004 (10)	−0.004 (10)	−0.004 (10)
Li4	0.035 (12)	0.035 (12)	0.035 (12)	0.000 (10)	0.000 (10)	0.000 (10)
B1	0.0112 (10)	0.0112 (10)	0.0112 (10)	−0.0005 (15)	−0.0005 (15)	−0.0005 (15)
B2	0.0119 (10)	0.0119 (10)	0.0119 (10)	−0.0001 (15)	−0.0001 (15)	−0.0001 (15)
Al1	0.0115 (6)	0.0119 (6)	0.0120 (6)	0.000	0.000	0.0003 (4)
O1	0.0185 (10)	0.0161 (9)	0.0164 (10)	0.0050 (6)	0.0069 (9)	0.0048 (9)
O2	0.0196 (11)	0.0164 (10)	0.0154 (9)	0.0071 (9)	0.0050 (6)	0.0048 (9)
Cl1	0.0774 (16)	0.0774 (16)	0.0774 (16)	0.000	0.000	0.000
Cl2	0.0786 (16)	0.0786 (16)	0.0786 (16)	0.000	0.000	0.000

### Lithium aluminoboracite (kjh0818yoshinoL14_0m_a_1) Geometric parameters (Å, º)

**Table:**

Li1—Li2^i^	0.48 (10)	Li3—O2^xxiii^	2.23 (3)
Li1—O1^ii^	2.053 (3)	Li3—O2^xxiv^	2.23 (3)
Li1—O1	2.053 (3)	Li3—Cl1	2.55 (6)
Li1—O2^iii^	2.143 (16)	Li3—O1^xxv^	2.824 (8)
Li1—O2^iv^	2.143 (16)	Li3—O1^xxvi^	2.824 (8)
Li1—Li3^v^	2.57 (4)	Li3—O1^vi^	2.824 (8)
Li1—Li3^vi^	2.57 (4)	Li4—O1^xxvii^	2.23 (3)
Li1—Li4^vii^	2.91 (4)	Li4—O1^xxviii^	2.23 (3)
Li1—Li4^viii^	2.91 (4)	Li4—O1^xxix^	2.23 (3)
Li1—Cl1	2.98 (6)	Li4—Cl2	2.55 (5)
Li1—Al1^ix^	3.256 (5)	Li4—O2^xxx^	2.826 (8)
Li1—Al1^x^	3.256 (5)	Li4—O2^xxxi^	2.826 (8)
Li2—O2^xi^	2.056 (4)	Li4—O2^xxxii^	2.826 (8)
Li2—O2^xii^	2.056 (4)	Li4—Al1^xxxiii^	2.91 (2)
Li2—O1^xiii^	2.132 (16)	Li4—Al1^xxxiv^	2.91 (2)
Li2—O1^xiv^	2.132 (16)	B1—O1	1.3693 (17)
Li2—Li4^xv^	2.60 (4)	B1—O1^ix^	1.3693 (17)
Li2—Li4^xvi^	2.60 (4)	B1—O1^x^	1.3693 (17)
Li2—Li3^xvii^	2.88 (4)	B2—O2^xxxv^	1.3702 (17)
Li2—Li3^xviii^	2.88 (4)	B2—O2^xii^	1.3702 (17)
Li2—Cl2	3.03 (6)	B2—O2^xxxvi^	1.3702 (17)
Li2—Al1^xix^	3.253 (4)	Al1—O1^xxxvii^	1.754 (2)
Li2—Al1^xx^	3.253 (4)	Al1—O2^xxxviii^	1.754 (2)
Li2—Al1^xxi^	3.253 (4)	Al1—O1^x^	1.754 (2)
Li3—O2^xxii^	2.23 (3)	Al1—O2^xxxix^	1.754 (2)
			
Li2^i^—Li1—O1^ii^	92.8 (16)	Li2^xvi^—Li4—Li2^xliii^	110.8 (14)
Li2^i^—Li1—O1	92.8 (16)	Li2^xlii^—Li4—Li2^xliii^	110.9 (14)
O1^ii^—Li1—O1	174 (3)	O1^xxvii^—Li4—O2^xxx^	157 (2)
Li2^i^—Li1—O2^iii^	73.1 (14)	O1^xxviii^—Li4—O2^xxx^	71.5 (2)
O1^ii^—Li1—O2^iii^	90.8 (4)	O1^xxix^—Li4—O2^xxx^	66.70 (16)
O1—Li1—O2^iii^	90.8 (4)	Cl2—Li4—O2^xxx^	82.1 (11)
Li2^i^—Li1—O2^iv^	73.1 (14)	Li2^xvi^—Li4—O2^xxx^	44.3 (2)
O1^ii^—Li1—O2^iv^	90.8 (4)	Li2^xlii^—Li4—O2^xxx^	74.0 (4)
O1—Li1—O2^iv^	90.8 (4)	Li2^xliii^—Li4—O2^xxx^	150 (2)
O2^iii^—Li1—O2^iv^	146 (3)	O1^xxvii^—Li4—O2^xxxi^	66.70 (16)
Li2^i^—Li1—Li3^v^	126.0 (15)	O1^xxviii^—Li4—O2^xxxi^	157 (2)
O1^ii^—Li1—Li3^v^	74.3 (8)	O1^xxix^—Li4—O2^xxxi^	71.5 (2)
O1—Li1—Li3^v^	102.3 (12)	Cl2—Li4—O2^xxxi^	82.1 (11)
O2^iii^—Li1—Li3^v^	55.4 (12)	Li2^xvi^—Li4—O2^xxxi^	150 (2)
O2^iv^—Li1—Li3^v^	155 (2)	Li2^xlii^—Li4—O2^xxxi^	44.3 (2)
Li2^i^—Li1—Li3^vi^	126.0 (15)	Li2^xliii^—Li4—O2^xxxi^	74.0 (4)
O1^ii^—Li1—Li3^vi^	102.3 (12)	O2^xxx^—Li4—O2^xxxi^	118.2 (5)
O1—Li1—Li3^vi^	74.3 (8)	O1^xxvii^—Li4—O2^xxxii^	71.5 (2)
O2^iii^—Li1—Li3^vi^	155 (2)	O1^xxviii^—Li4—O2^xxxii^	66.70 (16)
O2^iv^—Li1—Li3^vi^	55.4 (12)	O1^xxix^—Li4—O2^xxxii^	157 (2)
Li3^v^—Li1—Li3^vi^	108 (3)	Cl2—Li4—O2^xxxii^	82.1 (11)
Li2^i^—Li1—Li4^vii^	45.7 (13)	Li2^xvi^—Li4—O2^xxxii^	74.0 (4)
O1^ii^—Li1—Li4^vii^	136 (2)	Li2^xlii^—Li4—O2^xxxii^	150 (2)
O1—Li1—Li4^vii^	49.6 (13)	Li2^xliii^—Li4—O2^xxxii^	44.3 (2)
O2^iii^—Li1—Li4^vii^	65.9 (10)	O2^xxx^—Li4—O2^xxxii^	118.2 (5)
O2^iv^—Li1—Li4^vii^	90.1 (14)	O2^xxxi^—Li4—O2^xxxii^	118.2 (5)
Li3^v^—Li1—Li4^vii^	114.3 (10)	O1^xxvii^—Li4—Al1^xxxiii^	68.7 (7)
Li3^vi^—Li1—Li4^vii^	114.3 (10)	O1^xxviii^—Li4—Al1^xxxiii^	37.0 (3)
Li2^i^—Li1—Li4^viii^	45.7 (13)	O1^xxix^—Li4—Al1^xxxiii^	122 (2)
O1^ii^—Li1—Li4^viii^	49.6 (13)	Cl2—Li4—Al1^xxxiii^	114.3 (9)
O1—Li1—Li4^viii^	136 (2)	Li2^xvi^—Li4—Al1^xxxiii^	72.2 (6)
O2^iii^—Li1—Li4^viii^	90.1 (14)	Li2^xlii^—Li4—Al1^xxxiii^	174 (2)
O2^iv^—Li1—Li4^viii^	65.9 (10)	Li2^xliii^—Li4—Al1^xxxiii^	72.2 (6)
Li3^v^—Li1—Li4^viii^	114.3 (10)	O2^xxx^—Li4—Al1^xxxiii^	106.3 (4)
Li3^vi^—Li1—Li4^viii^	114.3 (10)	O2^xxxi^—Li4—Al1^xxxiii^	134.6 (7)
Li4^vii^—Li1—Li4^viii^	91 (3)	O2^xxxii^—Li4—Al1^xxxiii^	35.59 (12)
Li2^i^—Li1—Cl1	180.0	O1^xxvii^—Li4—Al1^xxxiv^	37.0 (3)
O1^ii^—Li1—Cl1	87.2 (16)	O1^xxviii^—Li4—Al1^xxxiv^	122 (2)
O1—Li1—Cl1	87.2 (16)	O1^xxix^—Li4—Al1^xxxiv^	68.7 (7)
O2^iii^—Li1—Cl1	106.9 (14)	Cl2—Li4—Al1^xxxiv^	114.3 (9)
O2^iv^—Li1—Cl1	106.9 (14)	Li2^xvi^—Li4—Al1^xxxiv^	174 (2)
Li3^v^—Li1—Cl1	54.0 (15)	Li2^xlii^—Li4—Al1^xxxiv^	72.2 (6)
Li3^vi^—Li1—Cl1	54.0 (15)	Li2^xliii^—Li4—Al1^xxxiv^	72.2 (6)
Li4^vii^—Li1—Cl1	134.3 (13)	O2^xxx^—Li4—Al1^xxxiv^	134.6 (7)
Li4^viii^—Li1—Cl1	134.3 (13)	O2^xxxi^—Li4—Al1^xxxiv^	35.59 (12)
Li2^i^—Li1—Al1^ix^	85.4 (10)	O2^xxxii^—Li4—Al1^xxxiv^	106.3 (4)
O1^ii^—Li1—Al1^ix^	152.41 (19)	Al1^xxxiii^—Li4—Al1^xxxiv^	104.3 (11)
O1—Li1—Al1^ix^	28.56 (17)	O1—B1—O1^ix^	119.972 (15)
O2^iii^—Li1—Al1^ix^	114.7 (7)	O1—B1—O1^x^	119.972 (15)
O2^iv^—Li1—Al1^ix^	62.3 (2)	O1^ix^—B1—O1^x^	119.972 (15)
Li3^v^—Li1—Al1^ix^	128.1 (12)	O2^xxxv^—B2—O2^xii^	119.982 (12)
Li3^vi^—Li1—Al1^ix^	58.5 (7)	O2^xxxv^—B2—O2^xxxvi^	119.982 (13)
Li4^vii^—Li1—Al1^ix^	55.9 (7)	O2^xii^—B2—O2^xxxvi^	119.982 (12)
Li4^viii^—Li1—Al1^ix^	116.6 (14)	O1^xxxvii^—Al1—O2^xxxviii^	107.12 (9)
Cl1—Li1—Al1^ix^	94.6 (10)	O1^xxxvii^—Al1—O1^x^	114.42 (15)
Li2^i^—Li1—Al1^x^	85.4 (10)	O2^xxxviii^—Al1—O1^x^	107.03 (8)
O1^ii^—Li1—Al1^x^	117.73 (11)	O1^xxxvii^—Al1—O2^xxxix^	107.03 (8)
O1—Li1—Al1^x^	62.78 (10)	O2^xxxviii^—Al1—O2^xxxix^	114.35 (15)
O2^iii^—Li1—Al1^x^	29.72 (10)	O1^x^—Al1—O2^xxxix^	107.12 (9)
O2^iv^—Li1—Al1^x^	145.3 (15)	O1^xxxvii^—Al1—Li4^xliv^	95.5 (7)
Li3^v^—Li1—Al1^x^	58.5 (7)	O2^xxxviii^—Al1—Li4^xliv^	153.8 (2)
Li3^vi^—Li1—Al1^x^	128.1 (12)	O1^x^—Al1—Li4^xliv^	49.8 (3)
Li4^vii^—Li1—Al1^x^	55.9 (7)	O2^xxxix^—Al1—Li4^xliv^	69.7 (8)
Li4^viii^—Li1—Al1^x^	116.6 (14)	O1^xxxvii^—Al1—Li4^xlv^	49.8 (3)
Cl1—Li1—Al1^x^	94.6 (10)	O2^xxxviii^—Al1—Li4^xlv^	69.7 (8)
Al1^ix^—Li1—Al1^x^	89.62 (17)	O1^x^—Al1—Li4^xlv^	95.5 (7)
O2^xi^—Li2—O2^xii^	172 (3)	O2^xxxix^—Al1—Li4^xlv^	153.8 (2)
O2^xi^—Li2—O1^xiii^	91.1 (4)	Li4^xliv^—Al1—Li4^xlv^	119.1 (19)
O2^xii^—Li2—O1^xiii^	91.1 (4)	O1^xxxvii^—Al1—Li3^ii^	153.8 (2)
O2^xi^—Li2—O1^xiv^	91.1 (4)	O2^xxxviii^—Al1—Li3^ii^	49.8 (3)
O2^xii^—Li2—O1^xiv^	91.1 (4)	O1^x^—Al1—Li3^ii^	69.5 (9)
O1^xiii^—Li2—O1^xiv^	148 (3)	O2^xxxix^—Al1—Li3^ii^	95.6 (7)
O2^xi^—Li2—Li4^xv^	101.4 (13)	Li4^xliv^—Al1—Li3^ii^	104.9 (7)
O2^xii^—Li2—Li4^xv^	73.7 (9)	Li4^xlv^—Al1—Li3^ii^	104.9 (7)
O1^xiii^—Li2—Li4^xv^	154 (2)	O1^xxxvii^—Al1—Li3^xli^	69.5 (9)
O1^xiv^—Li2—Li4^xv^	55.1 (12)	O2^xxxviii^—Al1—Li3^xli^	95.6 (7)
O2^xi^—Li2—Li4^xvi^	73.7 (9)	O1^x^—Al1—Li3^xli^	153.8 (2)
O2^xii^—Li2—Li4^xvi^	101.4 (13)	O2^xxxix^—Al1—Li3^xli^	49.8 (3)
O1^xiii^—Li2—Li4^xvi^	55.1 (12)	Li4^xliv^—Al1—Li3^xli^	104.9 (7)
O1^xiv^—Li2—Li4^xvi^	154 (2)	Li4^xlv^—Al1—Li3^xli^	104.9 (7)
Li4^xv^—Li2—Li4^xvi^	107 (3)	Li3^ii^—Al1—Li3^xli^	119 (2)
O2^xi^—Li2—Li3^xvii^	137 (2)	O1^xxxvii^—Al1—Li2^xlvi^	37.0 (5)
O2^xii^—Li2—Li3^xvii^	50.2 (14)	O2^xxxviii^—Al1—Li2^xlvi^	74.9 (9)
O1^xiii^—Li2—Li3^xvii^	66.6 (10)	O1^x^—Al1—Li2^xlvi^	143.0 (5)
O1^xiv^—Li2—Li3^xvii^	91.1 (15)	O2^xxxix^—Al1—Li2^xlvi^	105.1 (9)
Li4^xv^—Li2—Li3^xvii^	114.4 (10)	Li4^xliv^—Al1—Li2^xlvi^	130.5 (9)
Li4^xvi^—Li2—Li3^xvii^	114.4 (10)	Li4^xlv^—Al1—Li2^xlvi^	49.5 (9)
O2^xi^—Li2—Li3^xviii^	50.2 (13)	Li3^ii^—Al1—Li2^xlvi^	124.6 (9)
O2^xii^—Li2—Li3^xviii^	137 (2)	Li3^xli^—Al1—Li2^xlvi^	55.4 (9)
O1^xiii^—Li2—Li3^xviii^	91.1 (15)	O1^xxxvii^—Al1—Li2^xlvii^	98.7 (9)
O1^xiv^—Li2—Li3^xviii^	66.6 (10)	O2^xxxviii^—Al1—Li2^xlvii^	34.2 (3)
Li4^xv^—Li2—Li3^xviii^	114.4 (10)	O1^x^—Al1—Li2^xlvii^	81.3 (9)
Li4^xvi^—Li2—Li3^xviii^	114.4 (10)	O2^xxxix^—Al1—Li2^xlvii^	145.8 (3)
Li3^xvii^—Li2—Li3^xviii^	92 (3)	Li4^xliv^—Al1—Li2^xlvii^	130.5 (9)
O2^xi^—Li2—Cl2	86.0 (17)	Li4^xlv^—Al1—Li2^xlvii^	49.5 (9)
O2^xii^—Li2—Cl2	86.0 (17)	Li3^ii^—Al1—Li2^xlvii^	55.4 (9)
O1^xiii^—Li2—Cl2	105.9 (15)	Li3^xli^—Al1—Li2^xlvii^	124.6 (9)
O1^xiv^—Li2—Cl2	105.9 (15)	Li2^xlvi^—Al1—Li2^xlvii^	82 (2)
Li4^xv^—Li2—Cl2	53.3 (15)	O1^xxxvii^—Al1—Li2^xlviii^	81.3 (9)
Li4^xvi^—Li2—Cl2	53.3 (15)	O2^xxxviii^—Al1—Li2^xlviii^	145.8 (3)
Li3^xvii^—Li2—Cl2	133.8 (13)	O1^x^—Al1—Li2^xlviii^	98.7 (9)
Li3^xviii^—Li2—Cl2	133.8 (13)	O2^xxxix^—Al1—Li2^xlviii^	34.2 (3)
O2^xi^—Li2—Al1^xix^	117.76 (6)	Li4^xliv^—Al1—Li2^xlviii^	49.5 (9)
O2^xii^—Li2—Al1^xix^	62.84 (9)	Li4^xlv^—Al1—Li2^xlviii^	130.5 (9)
O1^xiii^—Li2—Al1^xix^	29.66 (11)	Li3^ii^—Al1—Li2^xlviii^	124.6 (9)
O1^xiv^—Li2—Al1^xix^	146.3 (15)	Li3^xli^—Al1—Li2^xlviii^	55.4 (9)
Li4^xv^—Li2—Al1^xix^	127.3 (13)	Li2^xlvi^—Al1—Li2^xlviii^	98 (2)
Li4^xvi^—Li2—Al1^xix^	58.3 (7)	Li2^xlvii^—Al1—Li2^xlviii^	179.99 (5)
Li3^xvii^—Li2—Al1^xix^	56.2 (7)	O1^xxxvii^—Al1—Li2^xlix^	143.0 (5)
Li3^xviii^—Li2—Al1^xix^	117.5 (15)	O2^xxxviii^—Al1—Li2^xlix^	105.1 (9)
Cl2—Li2—Al1^xix^	93.9 (10)	O1^x^—Al1—Li2^xlix^	37.0 (5)
O2^xi^—Li2—Al1^xx^	152.45 (6)	O2^xxxix^—Al1—Li2^xlix^	74.9 (9)
O2^xii^—Li2—Al1^xx^	28.68 (17)	Li4^xliv^—Al1—Li2^xlix^	49.5 (9)
O1^xiii^—Li2—Al1^xx^	115.2 (7)	Li4^xlv^—Al1—Li2^xlix^	130.5 (9)
O1^xiv^—Li2—Al1^xx^	62.41 (19)	Li3^ii^—Al1—Li2^xlix^	55.4 (9)
Li4^xv^—Li2—Al1^xx^	58.3 (7)	Li3^xli^—Al1—Li2^xlix^	124.6 (9)
Li4^xvi^—Li2—Al1^xx^	127.3 (13)	Li2^xlvi^—Al1—Li2^xlix^	180.0 (17)
Li3^xvii^—Li2—Al1^xx^	56.2 (7)	Li2^xlvii^—Al1—Li2^xlix^	98 (2)
Li3^xviii^—Li2—Al1^xx^	117.5 (15)	Li2^xlviii^—Al1—Li2^xlix^	82 (2)
Cl2—Li2—Al1^xx^	93.9 (10)	B1—O1—Al1^ix^	119.05 (13)
Al1^xix^—Li2—Al1^xx^	89.75 (15)	B1—O1—Li1	112.4 (12)
O2^xi^—Li2—Al1^xxi^	28.68 (17)	Al1^ix^—O1—Li1	117.4 (4)
O2^xii^—Li2—Al1^xxi^	152.45 (6)	B1—O1—Li2^i^	122.3 (12)
O1^xiii^—Li2—Al1^xxi^	62.41 (19)	Al1^ix^—O1—Li2^i^	113.4 (6)
O1^xiv^—Li2—Al1^xxi^	115.2 (7)	Li1—O1—Li2^i^	13 (3)
Li4^xv^—Li2—Al1^xxi^	127.3 (13)	B1—O1—Li4^vii^	123.9 (9)
Li4^xvi^—Li2—Al1^xxi^	58.3 (7)	Al1^ix^—O1—Li4^vii^	93.12 (8)
Li3^xvii^—Li2—Al1^xxi^	117.5 (15)	Li1—O1—Li4^vii^	85.7 (19)
Li3^xviii^—Li2—Al1^xxi^	56.2 (7)	Li2^i^—O1—Li4^vii^	73.2 (19)
Cl2—Li2—Al1^xxi^	93.9 (10)	B1—O1—Li3^vi^	102.2 (9)
Al1^xix^—Li2—Al1^xxi^	89.73 (15)	Al1^ix^—O1—Li3^vi^	74.9 (10)
Al1^xx^—Li2—Al1^xxi^	172 (2)	Li1—O1—Li3^vi^	61.3 (10)
O2^xxii^—Li3—O2^xxiii^	96.4 (16)	Li2^i^—O1—Li3^vi^	69.5 (10)
O2^xxii^—Li3—O2^xxiv^	96.4 (16)	Li4^vii^—O1—Li3^vi^	131.5 (12)
O2^xxiii^—Li3—O2^xxiv^	96.4 (16)	B1—O1—Li3	75.8 (3)
O2^xxii^—Li3—Cl1	120.6 (12)	Al1^ix^—O1—Li3	112.35 (10)
O2^xxiii^—Li3—Cl1	120.6 (12)	Li1—O1—Li3	50.3 (16)
O2^xxiv^—Li3—Cl1	120.6 (12)	Li2^i^—O1—Li3	63.2 (15)
O2^xxii^—Li3—Li1^vi^	108.9 (5)	Li4^vii^—O1—Li3	135.3 (9)
O2^xxiii^—Li3—Li1^vi^	140.8 (8)	Li3^vi^—O1—Li3	38.8 (10)
O2^xxiv^—Li3—Li1^vi^	52.5 (10)	B2^l^—O2—Al1^xxvi^	118.94 (13)
Cl1—Li3—Li1^vi^	71.3 (15)	B2^l^—O2—Li2^l^	113.5 (13)
O2^xxii^—Li3—Li1^xxvi^	52.4 (10)	Al1^xxvi^—O2—Li2^l^	117.1 (5)
O2^xxiii^—Li3—Li1^xxvi^	108.9 (5)	B2^l^—O2—Li1^li^	123.2 (11)
O2^xxiv^—Li3—Li1^xxvi^	140.8 (8)	Al1^xxvi^—O2—Li1^li^	113.0 (5)
Cl1—Li3—Li1^xxvi^	71.3 (15)	Li2^l^—O2—Li1^li^	13 (3)
Li1^vi^—Li3—Li1^xxvi^	110.2 (15)	B2^l^—O2—Li3^lii^	123.8 (10)
O2^xxii^—Li3—Li1^xxv^	140.8 (8)	Al1^xxvi^—O2—Li3^lii^	93.19 (8)
O2^xxiii^—Li3—Li1^xxv^	52.4 (10)	Li2^l^—O2—Li3^lii^	85 (2)
O2^xxiv^—Li3—Li1^xxv^	108.9 (5)	Li1^li^—O2—Li3^lii^	72.1 (18)
Cl1—Li3—Li1^xxv^	71.3 (15)	B2^l^—O2—Li4^liii^	102.4 (9)
Li1^vi^—Li3—Li1^xxv^	110.2 (15)	Al1^xxvi^—O2—Li4^liii^	74.7 (10)
Li1^xxvi^—Li3—Li1^xxv^	110.2 (15)	Li2^l^—O2—Li4^liii^	62.0 (11)
O2^xxii^—Li3—O1^xxv^	157 (2)	Li1^li^—O2—Li4^liii^	70.2 (10)
O2^xxiii^—Li3—O1^xxv^	71.5 (2)	Li3^lii^—O2—Li4^liii^	131.4 (12)
O2^xxiv^—Li3—O1^xxv^	66.68 (17)	B2^l^—O2—Li4	82.6 (3)
Cl1—Li3—O1^xxv^	82.2 (11)	Al1^xxvi^—O2—Li4	90.33 (9)
Li1^vi^—Li3—O1^xxv^	74.0 (4)	Li2^l^—O2—Li4	64.2 (14)
Li1^xxvi^—Li3—O1^xxv^	149 (2)	Li1^li^—O2—Li4	75.6 (13)
Li1^xxv^—Li3—O1^xxv^	44.4 (3)	Li3^lii^—O2—Li4	146.2 (12)
O2^xxii^—Li3—O1^xxvi^	71.5 (2)	Li4^liii^—O2—Li4	21.0 (10)
O2^xxiii^—Li3—O1^xxvi^	66.68 (17)	Li3—Cl1—Li3^ii^	109.468 (7)
O2^xxiv^—Li3—O1^xxvi^	157 (2)	Li3—Cl1—Li3^v^	109.473 (3)
Cl1—Li3—O1^xxvi^	82.2 (11)	Li3^ii^—Cl1—Li3^v^	109.473 (1)
Li1^vi^—Li3—O1^xxvi^	149 (2)	Li3—Cl1—Li3^vi^	109.473 (1)
Li1^xxvi^—Li3—O1^xxvi^	44.4 (3)	Li3^ii^—Cl1—Li3^vi^	109.5
Li1^xxv^—Li3—O1^xxvi^	73.9 (4)	Li3^v^—Cl1—Li3^vi^	109.468 (2)
O1^xxv^—Li3—O1^xxvi^	118.2 (5)	Li3—Cl1—Li1	125.266 (1)
O2^xxii^—Li3—O1^vi^	66.68 (17)	Li3^ii^—Cl1—Li1	125.266 (4)
O2^xxiii^—Li3—O1^vi^	157 (2)	Li3^v^—Cl1—Li1	54.734 (1)
O2^xxiv^—Li3—O1^vi^	71.5 (2)	Li3^vi^—Cl1—Li1	54.734 (2)
Cl1—Li3—O1^vi^	82.2 (11)	Li3—Cl1—Li1^xxvi^	54.736 (2)
Li1^vi^—Li3—O1^vi^	44.4 (3)	Li3^ii^—Cl1—Li1^xxvi^	125.264 (3)
Li1^xxvi^—Li3—O1^vi^	73.9 (4)	Li3^v^—Cl1—Li1^xxvi^	54.736 (2)
Li1^xxv^—Li3—O1^vi^	149 (2)	Li3^vi^—Cl1—Li1^xxvi^	125.264 (2)
O1^xxv^—Li3—O1^vi^	118.2 (5)	Li1—Cl1—Li1^xxvi^	90.000 (3)
O1^xxvi^—Li3—O1^vi^	118.2 (5)	Li3—Cl1—Li1^ix^	125.264 (5)
O2^xxii^—Li3—Li2^xl^	45.2 (8)	Li3^ii^—Cl1—Li1^ix^	54.736 (1)
O2^xxiii^—Li3—Li2^xl^	105.2 (6)	Li3^v^—Cl1—Li1^ix^	54.736 (2)
O2^xxiv^—Li3—Li2^xl^	137.0 (11)	Li3^vi^—Cl1—Li1^ix^	125.264 (4)
Cl1—Li3—Li2^xl^	79.1 (13)	Li1—Cl1—Li1^ix^	90.000 (3)
Li1^vi^—Li3—Li2^xl^	113.8 (9)	Li1^xxvi^—Cl1—Li1^ix^	90.0
Li1^xxvi^—Li3—Li2^xl^	7.8 (17)	Li3—Cl1—Li1^xxv^	54.736 (2)
Li1^xxv^—Li3—Li2^xl^	113.8 (9)	Li3^ii^—Cl1—Li1^xxv^	125.264 (1)
O1^xxv^—Li3—Li2^xl^	155.6 (18)	Li3^v^—Cl1—Li1^xxv^	125.264 (2)
O1^xxvi^—Li3—Li2^xl^	43.86 (15)	Li3^vi^—Cl1—Li1^xxv^	54.736 (2)
O1^vi^—Li3—Li2^xl^	74.5 (3)	Li1—Cl1—Li1^xxv^	90.000 (3)
O2^xxii^—Li3—Li2^xli^	105.2 (6)	Li1^xxvi^—Cl1—Li1^xxv^	90.0
O2^xxiii^—Li3—Li2^xli^	137.0 (11)	Li1^ix^—Cl1—Li1^xxv^	180.0
O2^xxiv^—Li3—Li2^xli^	45.2 (8)	Li3—Cl1—Li1^x^	125.264 (1)
Cl1—Li3—Li2^xli^	79.1 (13)	Li3^ii^—Cl1—Li1^x^	54.736 (1)
Li1^vi^—Li3—Li2^xli^	7.8 (17)	Li3^v^—Cl1—Li1^x^	125.264 (4)
Li1^xxvi^—Li3—Li2^xli^	113.8 (9)	Li3^vi^—Cl1—Li1^x^	54.736 (1)
Li1^xxv^—Li3—Li2^xli^	113.8 (9)	Li1—Cl1—Li1^x^	90.000 (4)
O1^xxv^—Li3—Li2^xli^	74.5 (3)	Li1^xxvi^—Cl1—Li1^x^	180.0
O1^xxvi^—Li3—Li2^xli^	155.6 (18)	Li1^ix^—Cl1—Li1^x^	90.000 (2)
O1^vi^—Li3—Li2^xli^	43.86 (15)	Li1^xxv^—Cl1—Li1^x^	90.0
Li2^xl^—Li3—Li2^xli^	116.5 (8)	Li3—Cl1—Li1^vi^	54.734 (2)
O1^xxvii^—Li4—O1^xxviii^	96.6 (15)	Li3^ii^—Cl1—Li1^vi^	54.734 (3)
O1^xxvii^—Li4—O1^xxix^	96.6 (15)	Li3^v^—Cl1—Li1^vi^	125.266 (2)
O1^xxviii^—Li4—O1^xxix^	96.6 (15)	Li3^vi^—Cl1—Li1^vi^	125.266 (1)
O1^xxvii^—Li4—Cl2	120.5 (12)	Li1—Cl1—Li1^vi^	180.0
O1^xxviii^—Li4—Cl2	120.5 (12)	Li1^xxvi^—Cl1—Li1^vi^	90.000 (5)
O1^xxix^—Li4—Cl2	120.5 (12)	Li1^ix^—Cl1—Li1^vi^	90.0
O1^xxvii^—Li4—Li2^xvi^	140.6 (8)	Li1^xxv^—Cl1—Li1^vi^	90.000 (4)
O1^xxviii^—Li4—Li2^xvi^	51.7 (10)	Li1^x^—Cl1—Li1^vi^	90.000 (1)
O1^xxix^—Li4—Li2^xvi^	108.6 (5)	Li4—Cl2—Li4^liv^	109.474 (11)
Cl2—Li4—Li2^xvi^	71.9 (15)	Li4—Cl2—Li4^xvi^	109.470 (2)
O1^xxvii^—Li4—Li2^xlii^	108.6 (5)	Li4^liv^—Cl2—Li4^xvi^	109.470 (3)
O1^xxviii^—Li4—Li2^xlii^	140.6 (8)	Li4—Cl2—Li4^xv^	109.470 (6)
O1^xxix^—Li4—Li2^xlii^	51.7 (10)	Li4^liv^—Cl2—Li4^xv^	109.5
Cl2—Li4—Li2^xlii^	71.9 (15)	Li4^xvi^—Cl2—Li4^xv^	109.474 (7)
Li2^xvi^—Li4—Li2^xlii^	110.8 (14)	Li4—Cl2—Li2	125.263 (4)
O1^xxvii^—Li4—Li2^xliii^	51.7 (10)	Li4^liv^—Cl2—Li2	125.263 (12)
O1^xxviii^—Li4—Li2^xliii^	108.6 (5)	Li4^xvi^—Cl2—Li2	54.737 (7)
O1^xxix^—Li4—Li2^xliii^	140.6 (8)	Li4^xv^—Cl2—Li2	54.737 (6)
Cl2—Li4—Li2^xliii^	71.9 (15)		

Symmetry codes: (i) −*x*+1/2, −*y*+1, *z*−1/2; (ii) *x*, −*y*+1/2, −*z*+1/2; (iii) −*x*, −*y*+1/2, *z*−1/2; (iv) −*x*, *y*, −*z*+1; (v) −*x*+1/2, *y*, −*z*+1/2; (vi) −*x*+1/2, −*y*+1/2, *z*; (vii) *x*−1, −*y*+1, −*z*+1; (viii) *x*−1, *y*−1/2, *z*−1/2; (ix) *y*, *z*, *x*; (x) *z*, *x*, *y*; (xi) *x*+1/2, −*y*+1, −*z*+3/2; (xii) *x*+1/2, *y*+1/2, *z*; (xiii) −*x*+1/2, *y*+1/2, −*z*+1; (xiv) −*x*+1/2, −*y*+1, *z*+1/2; (xv) −*x*+3/2, *y*, −*z*+3/2; (xvi) −*x*+3/2, −*y*+3/2, *z*; (xvii) *x*, −*y*+1, −*z*+1; (xviii) *x*, *y*+1/2, *z*+1/2; (xix) −*y*+1/2, *z*+1/2, −*x*+1; (xx) *z*+1/2, −*x*+1, −*y*+1/2; (xxi) *z*+1/2, *x*+1/2, *y*+1; (xxii) −*z*+1, *x*+1/2, −*y*+1/2; (xxiii) −*y*+1/2, −*z*+1, *x*+1/2; (xxiv) *x*+1/2, −*y*+1/2, −*z*+1; (xxv) −*y*+1/2, *z*, −*x*+1/2; (xxvi) *z*, −*x*+1/2, −*y*+1/2; (xxvii) −*z*+1, *x*+1, −*y*+1; (xxviii) *x*+1, −*y*+1, −*z*+1; (xxix) −*y*+1, −*z*+1, *x*+1; (xxx) −*x*+1, *y*+1/2, −*z*+3/2; (xxxi) *y*+1/2, −*z*+3/2, −*x*+1; (xxxii) −*z*+3/2, −*x*+1, *y*+1/2; (xxxiii) −*y*+1, *z*+1, −*x*+1; (xxxiv) −*x*+1, −*y*+1, *z*+1; (xxxv) *y*+1/2, *z*, *x*+1/2; (xxxvi) *z*, *x*+1/2, *y*+1/2; (xxxvii) *z*, −*x*, −*y*; (xxxviii) −*y*+1/2, *z*−1/2, −*x*; (xxxix) −*y*+1/2, −*z*+1/2, *x*; (xl) *z*−1/2, *x*, *y*−1/2; (xli) *x*, *y*−1/2, *z*−1/2; (xlii) −*y*+3/2, *z*, −*x*+3/2; (xliii) *z*, −*x*+3/2, −*y*+3/2; (xliv) −*x*+1, *y*−1, −*z*+1; (xlv) −*x*+1, −*y*+1, *z*−1; (xlvi) *z*−1/2, *x*−1/2, *y*−1; (xlvii) −*y*+1, *z*−1/2, −*x*+1/2; (xlviii) *y*−1/2, *z*−1, *x*−1/2; (xlix) *z*−1/2, −*x*+1/2, −*y*+1; (l) *x*−1/2, *y*−1/2, *z*; (li) −*x*, −*y*+1/2, *z*+1/2; (lii) *x*−1/2, −*y*+1/2, −*z*+1; (liii) −*x*+1, *y*−1/2, −*z*+3/2; (liv) *x*, −*y*+3/2, −*z*+3/2.

### Sodium aluminoboracite (kjh230804yoshinoN4_0m_a) Crystal data

**Table:**

Na_3.92_B_4_Al_3_O_12_Cl_0.92_	Cu *K*α radiation, λ = 1.54178 Å
*M**_r_* = 438.91	Cell parameters from 3423 reflections
Cubic, *F*43*c*	θ = 4.6–74.3°
*a* = 13.5904 (1) Å	µ = 6.78 mm^−^^1^
*V* = 2510.13 (6) Å^3^	*T* = 294 K
*Z* = 8	Plate, colorless
*F*(000) = 1728	0.08 × 0.06 × 0.04 mm
*D*_x_ = 2.323 Mg m^−^^3^	

### Sodium aluminoboracite (kjh230804yoshinoN4_0m_a) Data collection

**Table:**

Bruker D8 goniometer diffractometer	218 independent reflections
Radiation source: sealed tube	216 reflections with *I* > 2σ(*I*)
Detector resolution: 7.3910 pixels mm^-1^	*R*_int_ = 0.027
ω scans	θ_max_ = 74.3°, θ_min_ = 6.5°
Absorption correction: multi-scan (*SADABS*; Krause *et al.*, 2015)	*h* = −16→16
*T*_min_ = 0.66, *T*_max_ = 0.75	*k* = −16→16
4504 measured reflections	*l* = −16→14

### Sodium aluminoboracite (kjh230804yoshinoN4_0m_a) Refinement

**Table:**

Refinement on *F*^2^	1 restraint
Least-squares matrix: full	*w* = 1/[σ^2^(*F*_o_^2^) + (0.0311*P*)^2^ + 4.0293*P*] where *P* = (*F*_o_^2^ + 2*F*_c_^2^)/3
*R*[*F*^2^ > 2σ(*F*^2^)] = 0.022	(Δ/σ)_max_ < 0.001
*wR*(*F*^2^) = 0.055	Δρ_max_ = 0.15 e Å^−^^3^
*S* = 1.20	Δρ_min_ = −0.45 e Å^−^^3^
218 reflections	Absolute structure: Flack *x* determined using 89 quotients [(I+)-(I-)]/[(I+)+(I-)] (Parsons *et al.*, 2013)
27 parameters	Absolute structure parameter: 0.01 (3)

### Sodium aluminoboracite (kjh230804yoshinoN4_0m_a) Special details

**Table:**

Geometry. All esds (except the esd in the dihedral angle between two l.s. planes) are estimated using the full covariance matrix. The cell esds are taken into account individually in the estimation of esds in distances, angles and torsion angles; correlations between esds in cell parameters are only used when they are defined by crystal symmetry. An approximate (isotropic) treatment of cell esds is used for estimating esds involving l.s. planes.

### Sodium aluminoboracite (kjh230804yoshinoN4_0m_a) Fractional atomic coordinates and isotropic or equivalent isotropic displacement parameters (Å^2^)

**Table:**

	*x*	*y*	*z*	*U*_iso_*/*U*_eq_	Occ. (<1)
Na1	0.0340 (3)	0.250000	0.250000	0.0422 (14)	0.499 (4)
Na2	0.3606 (4)	0.3606 (4)	0.3606 (4)	0.017 (2)	0.231 (6)
B1	0.1039 (2)	0.1039 (2)	0.1039 (2)	0.0143 (9)	
Al1	0.250000	0.000000	0.000000	0.0095 (4)	
O1	0.03513 (13)	0.10047 (12)	0.17745 (12)	0.0176 (5)	
Cl1	0.250000	0.250000	0.250000	0.0350 (9)	0.920 (10)

### Sodium aluminoboracite (kjh230804yoshinoN4_0m_a) Atomic displacement parameters (Å^2^)

**Table:**

	*U*^11^	*U*^22^	*U*^33^	*U*^12^	*U*^13^	*U*^23^
Na1	0.096 (4)	0.0127 (18)	0.0182 (19)	0.000	0.000	−0.0066 (17)
Na2	0.017 (2)	0.017 (2)	0.017 (2)	−0.0006 (18)	−0.0006 (18)	−0.0006 (18)
B1	0.0143 (9)	0.0143 (9)	0.0143 (9)	0.0015 (12)	0.0015 (12)	0.0015 (12)
Al1	0.0096 (6)	0.0095 (5)	0.0095 (5)	0.000	0.000	0.000
O1	0.0189 (9)	0.0149 (8)	0.0188 (8)	0.0060 (6)	0.0102 (8)	0.0061 (7)
Cl1	0.0350 (9)	0.0350 (9)	0.0350 (9)	0.000	0.000	0.000

### Sodium aluminoboracite (kjh230804yoshinoN4_0m_a) Geometric parameters (Å, º)

**Table:**

Na1—Na1^i^	0.923 (8)	Na2—O1^x^	2.484 (5)
Na1—O1^ii^	2.2588 (16)	Na2—Cl1	2.605 (9)
Na1—O1	2.2588 (16)	Na2—O1^xi^	2.913 (2)
Na1—O1^iii^	2.446 (2)	Na2—O1^v^	2.913 (2)
Na1—O1^i^	2.446 (2)	Na2—O1^xii^	2.913 (2)
Na1—Na2^iv^	2.564 (4)	Na2—Al1^xiii^	3.072 (4)
Na1—Na2^v^	2.564 (4)	Na2—Al1^xiv^	3.072 (4)
Na1—Cl1	2.936 (4)	B1—O1^xv^	1.3692 (15)
Na1—B1^ii^	2.965 (3)	B1—O1^xvi^	1.3692 (15)
Na1—B1	2.965 (3)	B1—O1	1.3693 (15)
Na1—Na2^vi^	3.174 (3)	Al1—O1^xvii^	1.7506 (16)
Na1—Na2^vii^	3.174 (3)	Al1—O1^xviii^	1.7506 (16)
Na2—O1^viii^	2.484 (5)	Al1—O1^xvi^	1.7506 (16)
Na2—O1^ix^	2.484 (5)	Al1—O1^xix^	1.7506 (16)
			
Na1^i^—Na1—O1^ii^	90.40 (11)	O1—B1—Na1	46.75 (14)
Na1^i^—Na1—O1	90.40 (11)	O1^xv^—B1—Na1^xv^	46.75 (14)
O1^ii^—Na1—O1	179.2 (2)	O1^xvi^—B1—Na1^xv^	87.53 (15)
Na1^i^—Na1—O1^iii^	67.42 (9)	O1—B1—Na1^xv^	135.5 (3)
O1^ii^—Na1—O1^iii^	90.15 (4)	Na1—B1—Na1^xv^	88.88 (15)
O1—Na1—O1^iii^	90.15 (4)	O1^xv^—B1—Na1^xvi^	135.5 (3)
Na1^i^—Na1—O1^i^	67.42 (9)	O1^xvi^—B1—Na1^xvi^	46.75 (14)
O1^ii^—Na1—O1^i^	90.15 (4)	O1—B1—Na1^xvi^	87.53 (15)
O1—Na1—O1^i^	90.15 (4)	Na1—B1—Na1^xvi^	88.88 (15)
O1^iii^—Na1—O1^i^	134.84 (18)	Na1^xv^—B1—Na1^xvi^	88.88 (15)
Na1^i^—Na1—Na2^iv^	124.0 (2)	O1^xvii^—Al1—O1^xviii^	108.49 (6)
O1^ii^—Na1—Na2^iv^	74.01 (7)	O1^xvii^—Al1—O1^xvi^	111.44 (11)
O1—Na1—Na2^iv^	105.53 (9)	O1^xviii^—Al1—O1^xvi^	108.49 (6)
O1^iii^—Na1—Na2^iv^	59.39 (18)	O1^xvii^—Al1—O1^xix^	108.49 (6)
O1^i^—Na1—Na2^iv^	159.73 (13)	O1^xviii^—Al1—O1^xix^	111.44 (11)
Na1^i^—Na1—Na2^v^	124.0 (2)	O1^xvi^—Al1—O1^xix^	108.49 (6)
O1^ii^—Na1—Na2^v^	105.53 (9)	O1^xvii^—Al1—Na2^ii^	157.64 (7)
O1—Na1—Na2^v^	74.01 (7)	O1^xviii^—Al1—Na2^ii^	53.95 (9)
O1^iii^—Na1—Na2^v^	159.73 (13)	O1^xvi^—Al1—Na2^ii^	68.08 (15)
O1^i^—Na1—Na2^v^	59.39 (18)	O1^xix^—Al1—Na2^ii^	92.12 (11)
Na2^iv^—Na1—Na2^v^	112.1 (4)	O1^xvii^—Al1—Na2^xx^	68.08 (15)
Na1^i^—Na1—Cl1	180.0	O1^xviii^—Al1—Na2^xx^	92.12 (11)
O1^ii^—Na1—Cl1	89.60 (11)	O1^xvi^—Al1—Na2^xx^	157.64 (7)
O1—Na1—Cl1	89.60 (11)	O1^xix^—Al1—Na2^xx^	53.95 (9)
O1^iii^—Na1—Cl1	112.58 (9)	Na2^ii^—Al1—Na2^xx^	121.4 (3)
O1^i^—Na1—Cl1	112.58 (9)	O1^xvii^—Al1—Na2^xxi^	92.12 (11)
Na2^iv^—Na1—Cl1	56.0 (2)	O1^xviii^—Al1—Na2^xxi^	157.64 (7)
Na2^v^—Na1—Cl1	56.0 (2)	O1^xvi^—Al1—Na2^xxi^	53.95 (9)
Na1^i^—Na1—B1^ii^	108.69 (11)	O1^xix^—Al1—Na2^xxi^	68.08 (15)
O1^ii^—Na1—B1^ii^	26.20 (7)	Na2^ii^—Al1—Na2^xxi^	103.87 (13)
O1—Na1—B1^ii^	153.23 (14)	Na2^xx^—Al1—Na2^xxi^	103.87 (13)
O1^iii^—Na1—B1^ii^	114.17 (5)	O1^xvii^—Al1—Na2^xxii^	53.95 (9)
O1^i^—Na1—B1^ii^	80.59 (6)	O1^xviii^—Al1—Na2^xxii^	68.08 (15)
Na2^iv^—Na1—B1^ii^	79.69 (9)	O1^xvi^—Al1—Na2^xxii^	92.12 (11)
Na2^v^—Na1—B1^ii^	79.69 (9)	O1^xix^—Al1—Na2^xxii^	157.64 (7)
Cl1—Na1—B1^ii^	71.31 (11)	Na2^ii^—Al1—Na2^xxii^	103.87 (13)
Na1^i^—Na1—B1	108.68 (11)	Na2^xx^—Al1—Na2^xxii^	103.87 (13)
O1^ii^—Na1—B1	153.23 (14)	Na2^xxi^—Al1—Na2^xxii^	121.4 (3)
O1—Na1—B1	26.20 (7)	O1^xvii^—Al1—Na1^xxiii^	67.96 (7)
O1^iii^—Na1—B1	80.59 (6)	O1^xviii^—Al1—Na1^xxiii^	137.41 (6)
O1^i^—Na1—B1	114.17 (5)	O1^xvi^—Al1—Na1^xxiii^	112.04 (7)
Na2^iv^—Na1—B1	79.69 (9)	O1^xix^—Al1—Na1^xxiii^	42.59 (6)
Na2^v^—Na1—B1	79.69 (9)	Na2^ii^—Al1—Na1^xxiii^	133.95 (9)
Cl1—Na1—B1	71.32 (11)	Na2^xx^—Al1—Na1^xxiii^	46.05 (9)
B1^ii^—Na1—B1	142.6 (2)	Na2^xxi^—Al1—Na1^xxiii^	58.13 (7)
Na1^i^—Na1—Na2^vi^	42.07 (17)	Na2^xxii^—Al1—Na1^xxiii^	121.87 (7)
O1^ii^—Na1—Na2^vi^	129.7 (2)	O1^xvii^—Al1—Na1^xvii^	35.94 (5)
O1—Na1—Na2^vi^	51.10 (16)	O1^xviii^—Al1—Na1^xvii^	80.49 (8)
O1^iii^—Na1—Na2^vi^	60.81 (7)	O1^xvi^—Al1—Na1^xvii^	144.06 (5)
O1^i^—Na1—Na2^vi^	85.28 (12)	O1^xix^—Al1—Na1^xvii^	99.51 (8)
Na2^iv^—Na1—Na2^vi^	114.50 (19)	Na2^ii^—Al1—Na1^xvii^	133.95 (9)
Na2^v^—Na1—Na2^vi^	114.50 (19)	Na2^xx^—Al1—Na1^xvii^	46.05 (9)
Cl1—Na1—Na2^vi^	137.93 (17)	Na2^xxi^—Al1—Na1^xvii^	121.87 (7)
B1^ii^—Na1—Na2^vi^	150.8 (2)	Na2^xxii^—Al1—Na1^xvii^	58.13 (7)
B1—Na1—Na2^vi^	66.6 (2)	Na1^xxiii^—Al1—Na1^xvii^	74.52 (12)
Na1^i^—Na1—Na2^vii^	42.07 (17)	O1^xvii^—Al1—Na1^xvi^	144.06 (5)
O1^ii^—Na1—Na2^vii^	51.10 (16)	O1^xviii^—Al1—Na1^xvi^	99.51 (8)
O1—Na1—Na2^vii^	129.7 (2)	O1^xvi^—Al1—Na1^xvi^	35.94 (5)
O1^iii^—Na1—Na2^vii^	85.28 (12)	O1^xix^—Al1—Na1^xvi^	80.49 (8)
O1^i^—Na1—Na2^vii^	60.81 (7)	Na2^ii^—Al1—Na1^xvi^	46.05 (9)
Na2^iv^—Na1—Na2^vii^	114.50 (19)	Na2^xx^—Al1—Na1^xvi^	133.95 (9)
Na2^v^—Na1—Na2^vii^	114.50 (19)	Na2^xxi^—Al1—Na1^xvi^	58.13 (7)
Cl1—Na1—Na2^vii^	137.93 (17)	Na2^xxii^—Al1—Na1^xvi^	121.87 (7)
B1^ii^—Na1—Na2^vii^	66.6 (2)	Na1^xxiii^—Al1—Na1^xvi^	105.48 (12)
B1—Na1—Na2^vii^	150.8 (2)	Na1^xvii^—Al1—Na1^xvi^	180.00 (12)
Na2^vi^—Na1—Na2^vii^	84.1 (3)	O1^xvii^—Al1—Na1^xv^	112.04 (7)
O1^viii^—Na2—O1^ix^	92.3 (2)	O1^xviii^—Al1—Na1^xv^	42.59 (6)
O1^viii^—Na2—O1^x^	92.3 (2)	O1^xvi^—Al1—Na1^xv^	67.96 (7)
O1^ix^—Na2—O1^x^	92.3 (2)	O1^xix^—Al1—Na1^xv^	137.41 (6)
O1^viii^—Na2—Na1^xi^	108.59 (7)	Na2^ii^—Al1—Na1^xv^	46.05 (9)
O1^ix^—Na2—Na1^xi^	143.23 (13)	Na2^xx^—Al1—Na1^xv^	133.95 (9)
O1^x^—Na2—Na1^xi^	57.95 (8)	Na2^xxi^—Al1—Na1^xv^	121.87 (7)
O1^viii^—Na2—Na1^xii^	57.95 (8)	Na2^xxii^—Al1—Na1^xv^	58.13 (7)
O1^ix^—Na2—Na1^xii^	108.59 (7)	Na1^xxiii^—Al1—Na1^xv^	180.0
O1^x^—Na2—Na1^xii^	143.23 (13)	Na1^xvii^—Al1—Na1^xv^	105.48 (12)
Na1^xi^—Na2—Na1^xii^	108.1 (2)	Na1^xvi^—Al1—Na1^xv^	74.52 (12)
O1^viii^—Na2—Na1^v^	143.23 (13)	B1—O1—Al1^xv^	128.59 (12)
O1^ix^—Na2—Na1^v^	57.95 (8)	B1—O1—Na1	107.1 (2)
O1^x^—Na2—Na1^v^	108.59 (7)	Al1^xv^—O1—Na1	117.00 (8)
Na1^xi^—Na2—Na1^v^	108.1 (2)	B1—O1—Na1^i^	121.89 (16)
Na1^xii^—Na2—Na1^v^	108.1 (2)	Al1^xv^—O1—Na1^i^	108.44 (8)
O1^viii^—Na2—Cl1	123.62 (17)	Na1—O1—Na1^i^	22.18 (18)
O1^ix^—Na2—Cl1	123.62 (17)	B1—O1—Na2^vi^	119.5 (2)
O1^x^—Na2—Cl1	123.62 (17)	Al1^xv^—O1—Na2^vi^	91.32 (7)
Na1^xi^—Na2—Cl1	69.2 (2)	Na1—O1—Na2^vi^	83.85 (18)
Na1^xii^—Na2—Cl1	69.2 (2)	Na1^i^—O1—Na2^vi^	62.66 (18)
Na1^v^—Na2—Cl1	69.2 (2)	B1—O1—Na2^v^	106.6 (3)
O1^viii^—Na2—O1^xi^	62.95 (7)	Al1^xv^—O1—Na2^v^	78.04 (18)
O1^ix^—Na2—O1^xi^	151.5 (3)	Na1—O1—Na2^v^	57.80 (10)
O1^x^—Na2—O1^xi^	75.84 (7)	Na1^i^—O1—Na2^v^	72.03 (7)
Na1^xi^—Na2—O1^xi^	48.20 (5)	Na2^vi^—O1—Na2^v^	127.14 (10)
Na1^xii^—Na2—O1^xi^	71.49 (6)	Na2^ii^—Cl1—Na2^iv^	109.5
Na1^v^—Na2—O1^xi^	150.4 (3)	Na2^ii^—Cl1—Na2^v^	109.5
Cl1—Na2—O1^xi^	83.80 (18)	Na2^iv^—Cl1—Na2^v^	109.5
O1^viii^—Na2—O1^v^	151.5 (3)	Na2^ii^—Cl1—Na2	109.471 (1)
O1^ix^—Na2—O1^v^	75.84 (7)	Na2^iv^—Cl1—Na2	109.471 (1)
O1^x^—Na2—O1^v^	62.95 (7)	Na2^v^—Cl1—Na2	109.5
Na1^xi^—Na2—O1^v^	71.49 (6)	Na2^ii^—Cl1—Na1	125.3
Na1^xii^—Na2—O1^v^	150.4 (3)	Na2^iv^—Cl1—Na1	54.7
Na1^v^—Na2—O1^v^	48.20 (5)	Na2^v^—Cl1—Na1	54.7
Cl1—Na2—O1^v^	83.80 (18)	Na2—Cl1—Na1	125.3
O1^xi^—Na2—O1^v^	118.85 (6)	Na2^ii^—Cl1—Na1^xi^	125.3
O1^viii^—Na2—O1^xii^	75.84 (7)	Na2^iv^—Cl1—Na1^xi^	54.7
O1^ix^—Na2—O1^xii^	62.95 (7)	Na2^v^—Cl1—Na1^xi^	125.3
O1^x^—Na2—O1^xii^	151.5 (3)	Na2—Cl1—Na1^xi^	54.7
Na1^xi^—Na2—O1^xii^	150.4 (3)	Na1—Cl1—Na1^xi^	90.0
Na1^xii^—Na2—O1^xii^	48.20 (5)	Na2^ii^—Cl1—Na1^xv^	54.7
Na1^v^—Na2—O1^xii^	71.49 (6)	Na2^iv^—Cl1—Na1^xv^	54.7
Cl1—Na2—O1^xii^	83.80 (18)	Na2^v^—Cl1—Na1^xv^	125.3
O1^xi^—Na2—O1^xii^	118.85 (6)	Na2—Cl1—Na1^xv^	125.3
O1^v^—Na2—O1^xii^	118.85 (6)	Na1—Cl1—Na1^xv^	90.0
O1^viii^—Na2—Al1^xiii^	117.7 (3)	Na1^xi^—Cl1—Na1^xv^	90.0
O1^ix^—Na2—Al1^xiii^	69.15 (13)	Na2^ii^—Cl1—Na1^xii^	125.3
O1^x^—Na2—Al1^xiii^	34.73 (6)	Na2^iv^—Cl1—Na1^xii^	125.3
Na1^xi^—Na2—Al1^xiii^	74.34 (5)	Na2^v^—Cl1—Na1^xii^	54.7
Na1^xii^—Na2—Al1^xiii^	175.4 (3)	Na2—Cl1—Na1^xii^	54.7
Na1^v^—Na2—Al1^xiii^	74.34 (5)	Na1—Cl1—Na1^xii^	90.0
Cl1—Na2—Al1^xiii^	115.42 (15)	Na1^xi^—Cl1—Na1^xii^	90.0
O1^xi^—Na2—Al1^xiii^	108.42 (8)	Na1^xv^—Cl1—Na1^xii^	180.0
O1^v^—Na2—Al1^xiii^	33.89 (4)	Na2^ii^—Cl1—Na1^v^	54.7
O1^xii^—Na2—Al1^xiii^	130.79 (12)	Na2^iv^—Cl1—Na1^v^	125.3
O1^viii^—Na2—Al1^xiv^	69.15 (13)	Na2^v^—Cl1—Na1^v^	125.3
O1^ix^—Na2—Al1^xiv^	34.73 (6)	Na2—Cl1—Na1^v^	54.7
O1^x^—Na2—Al1^xiv^	117.7 (3)	Na1—Cl1—Na1^v^	180.0
Na1^xi^—Na2—Al1^xiv^	175.4 (3)	Na1^xi^—Cl1—Na1^v^	90.0
Na1^xii^—Na2—Al1^xiv^	74.34 (5)	Na1^xv^—Cl1—Na1^v^	90.0
Na1^v^—Na2—Al1^xiv^	74.34 (5)	Na1^xii^—Cl1—Na1^v^	90.0
Cl1—Na2—Al1^xiv^	115.42 (15)	Na2^ii^—Cl1—Na1^xvi^	54.7
O1^xi^—Na2—Al1^xiv^	130.79 (12)	Na2^iv^—Cl1—Na1^xvi^	125.3
O1^v^—Na2—Al1^xiv^	108.42 (8)	Na2^v^—Cl1—Na1^xvi^	54.7
O1^xii^—Na2—Al1^xiv^	33.89 (4)	Na2—Cl1—Na1^xvi^	125.3
Al1^xiii^—Na2—Al1^xiv^	102.92 (18)	Na1—Cl1—Na1^xvi^	90.0
O1^xv^—B1—O1^xvi^	119.993 (7)	Na1^xi^—Cl1—Na1^xvi^	180.0
O1^xv^—B1—O1	119.993 (7)	Na1^xv^—Cl1—Na1^xvi^	90.0
O1^xvi^—B1—O1	119.993 (7)	Na1^xii^—Cl1—Na1^xvi^	90.0
O1^xv^—B1—Na1	87.53 (15)	Na1^v^—Cl1—Na1^xvi^	90.0
O1^xvi^—B1—Na1	135.5 (3)		

Symmetry codes: (i) −*x*, *z*, −*y*+1/2; (ii) *x*, −*y*+1/2, −*z*+1/2; (iii) −*x*, −*z*+1/2, *y*; (iv) −*x*+1/2, *y*, −*z*+1/2; (v) −*x*+1/2, −*y*+1/2, *z*; (vi) *y*−1/2, −*x*+1/2, −*z*+1/2; (vii) *y*−1/2, *x*, *z*; (viii) −*z*+1/2, −*y*+1/2, *x*+1/2; (ix) *x*+1/2, −*z*+1/2, −*y*+1/2; (x) −*y*+1/2, *x*+1/2, −*z*+1/2; (xi) *z*, −*x*+1/2, −*y*+1/2; (xii) −*y*+1/2, *z*, −*x*+1/2; (xiii) *y*+1/2, *z*+1/2, *x*; (xiv) *z*+1/2, *x*, *y*+1/2; (xv) *y*, *z*, *x*; (xvi) *z*, *x*, *y*; (xvii) *z*, −*x*, −*y*; (xviii) −*z*+1/2, *y*, −*x*; (xix) −*z*+1/2, −*y*, *x*; (xx) *x*, *y*−1/2, *z*−1/2; (xxi) −*y*+1/2, *x*−1/2, −*z*+1/2; (xxii) −*y*+1/2, −*x*+1/2, *z*−1/2; (xxiii) −*y*+1/2, *z*−1/2, −*x*.

### Sodium aluminoboracite (kjh230804yoshinoN4_0m_a_1) Crystal data

**Table:**

Na_3.92_B_4_Al_3_O_12_Cl_0.92_	Cu *K*α radiation, λ = 1.54178 Å
*M**_r_* = 438.91	Cell parameters from 3423 reflections
Cubic, *F*23	θ = 4.6–74.3°
*a* = 13.5904 (1) Å	µ = 6.78 mm^−^^1^
*V* = 2510.13 (6) Å^3^	*T* = 294 K
*Z* = 8	Plate, colorless
*F*(000) = 1728	0.08 × 0.06 × 0.04 mm
*D*_x_ = 2.323 Mg m^−^^3^	

### Sodium aluminoboracite (kjh230804yoshinoN4_0m_a_1) Data collection

**Table:**

Bruker D8 goniometer diffractometer	437 independent reflections
Radiation source: sealed tube	355 reflections with *I* > 2σ(*I*)
Detector resolution: 7.3910 pixels mm^-1^	*R*_int_ = 0.028
ω scans	θ_max_ = 74.3°, θ_min_ = 5.6°
Absorption correction: multi-scan (*SADABS*; Krause *et al.*, 2015)	*h* = −16→16
*T*_min_ = 0.66, *T*_max_ = 0.75	*k* = −16→16
5514 measured reflections	*l* = −16→14

### Sodium aluminoboracite (kjh230804yoshinoN4_0m_a_1) Refinement

**Table:**

Refinement on *F*^2^	1 restraint
Least-squares matrix: full	*w* = 1/[σ^2^(*F*_o_^2^) + (0.0396*P*)^2^ + 2.0455*P*] where *P* = (*F*_o_^2^ + 2*F*_c_^2^)/3
*R*[*F*^2^ > 2σ(*F*^2^)] = 0.024	(Δ/σ)_max_ = 0.006
*wR*(*F*^2^) = 0.066	Δρ_max_ = 0.17 e Å^−^^3^
*S* = 1.12	Δρ_min_ = −0.58 e Å^−^^3^
437 reflections	Absolute structure: Flack *x* determined using 142 quotients [(I+)-(I-)]/[(I+)+(I-)] (Parsons *et al.*, 2013).
52 parameters	Absolute structure parameter: 0.01 (2)

### Sodium aluminoboracite (kjh230804yoshinoN4_0m_a_1) Special details

**Table:**

Geometry. All esds (except the esd in the dihedral angle between two l.s. planes) are estimated using the full covariance matrix. The cell esds are taken into account individually in the estimation of esds in distances, angles and torsion angles; correlations between esds in cell parameters are only used when they are defined by crystal symmetry. An approximate (isotropic) treatment of cell esds is used for estimating esds involving l.s. planes.

### Sodium aluminoboracite (kjh230804yoshinoN4_0m_a_1) Fractional atomic coordinates and isotropic or equivalent isotropic displacement parameters (Å^2^)

**Table:**

	*x*	*y*	*z*	*U*_iso_*/*U*_eq_	Occ. (<1)
Na1	0.0348 (5)	0.250000	0.250000	0.0429 (12)	0.5
Na2	0.5331 (5)	0.750000	0.750000	0.0430 (12)	0.5
Na3	0.3604 (4)	0.3604 (4)	0.3604 (4)	0.015 (2)	0.226 (7)
Na4	0.8610 (4)	0.8610 (4)	0.8610 (4)	0.020 (2)	0.235 (7)
B1	0.1039 (3)	0.1039 (3)	0.1039 (3)	0.0149 (10)	
B2	0.6039 (3)	0.6039 (3)	0.6039 (3)	0.0138 (10)	
Al1	0.25008 (10)	0.000000	0.000000	0.0099 (3)	
O1	0.03517 (13)	0.10051 (12)	0.17740 (13)	0.0181 (5)	
O2	0.03517 (13)	0.17741 (13)	0.60039 (12)	0.0182 (5)	
Cl1	0.250000	0.250000	0.250000	0.0359 (9)	0.921 (9)
Cl2	0.750000	0.750000	0.750000	0.0350 (9)	0.921 (9)

### Sodium aluminoboracite (kjh230804yoshinoN4_0m_a_1) Atomic displacement parameters (Å^2^)

**Table:**

	*U*^11^	*U*^22^	*U*^33^	*U*^12^	*U*^13^	*U*^23^
Na1	0.092 (4)	0.0156 (18)	0.0210 (19)	0.000	0.000	−0.0062 (17)
Na2	0.100 (4)	0.0169 (18)	0.0118 (17)	0.000	0.000	−0.0067 (16)
Na3	0.015 (2)	0.015 (2)	0.015 (2)	−0.0008 (19)	−0.0008 (19)	−0.0008 (19)
Na4	0.020 (2)	0.020 (2)	0.020 (2)	−0.002 (2)	−0.002 (2)	−0.002 (2)
B1	0.0149 (10)	0.0149 (10)	0.0149 (10)	0.0013 (13)	0.0013 (13)	0.0013 (13)
B2	0.0138 (10)	0.0138 (10)	0.0138 (10)	0.0030 (13)	0.0030 (13)	0.0030 (13)
Al1	0.0100 (5)	0.0099 (5)	0.0099 (5)	0.000	0.000	−0.0001 (4)
O1	0.0192 (9)	0.0153 (8)	0.0198 (10)	0.0061 (7)	0.0107 (8)	0.0059 (8)
O2	0.0194 (9)	0.0196 (10)	0.0156 (8)	0.0105 (8)	0.0056 (7)	0.0070 (8)
Cl1	0.0359 (9)	0.0359 (9)	0.0359 (9)	0.000	0.000	0.000
Cl2	0.0350 (9)	0.0350 (9)	0.0350 (9)	0.000	0.000	0.000

### Sodium aluminoboracite (kjh230804yoshinoN4_0m_a_1) Geometric parameters (Å, º)

**Table:**

Na1—Na2^i^	0.922 (6)	Na3—Cl1	2.599 (10)
Na1—O1^ii^	2.2585 (18)	Na3—O1^xxi^	2.912 (2)
Na1—O1	2.2585 (18)	Na3—O1^xxii^	2.912 (2)
Na1—O2^iii^	2.452 (3)	Na3—O1^vi^	2.912 (2)
Na1—O2^iv^	2.452 (3)	Na3—Al1^xvii^	3.074 (4)
Na1—Na3^v^	2.556 (5)	Na3—Al1^xxiii^	3.074 (4)
Na1—Na3^vi^	2.556 (5)	Na4—O1^xxiv^	2.480 (6)
Na1—Cl1	2.925 (6)	Na4—O1^xxv^	2.480 (6)
Na1—B1^ii^	2.961 (4)	Na4—O1^xxvi^	2.480 (6)
Na1—B1	2.961 (4)	Na4—Cl2	2.612 (10)
Na1—Na4^vii^	3.182 (5)	Na4—O2^xxvii^	2.914 (2)
Na1—Na4^viii^	3.182 (5)	Na4—O2^xxviii^	2.914 (2)
Na2—O2^ix^	2.2602 (18)	Na4—O2^xxix^	2.914 (2)
Na2—O2^x^	2.2602 (18)	Na4—Al1^xxx^	3.069 (4)
Na2—O1^xi^	2.442 (3)	Na4—Al1^xxxi^	3.069 (4)
Na2—O1^xii^	2.442 (3)	B1—O1^xxxii^	1.3684 (16)
Na2—Na4^xiii^	2.573 (5)	B1—O1^xxxiii^	1.3684 (16)
Na2—Na4^xiv^	2.573 (5)	B1—O1	1.3684 (16)
Na2—Cl2	2.948 (6)	B2—O2^xxxiv^	1.3685 (16)
Na2—B2^xv^	2.969 (4)	B2—O2^xxxv^	1.3684 (16)
Na2—B2	2.969 (4)	B2—O2^x^	1.3684 (16)
Na2—Na3^xvi^	3.164 (5)	Al1—O2^xxxvi^	1.7495 (18)
Na2—Na3^xvii^	3.164 (5)	Al1—O2^xxxvii^	1.7495 (18)
Na3—O2^xviii^	2.488 (6)	Al1—O1^xxxviii^	1.7522 (18)
Na3—O2^xix^	2.488 (6)	Al1—O1^xxxii^	1.7522 (18)
Na3—O2^xx^	2.488 (6)		
			
Na2^i^—Na1—O1^ii^	90.14 (17)	Cl2—Na4—O2^xxix^	83.7 (2)
Na2^i^—Na1—O1	90.14 (17)	O2^xxvii^—Na4—O2^xxix^	118.80 (8)
O1^ii^—Na1—O1	179.7 (3)	O2^xxviii^—Na4—O2^xxix^	118.80 (8)
Na2^i^—Na1—O2^iii^	67.19 (14)	O1^xxiv^—Na4—Al1^xxx^	118.0 (4)
O1^ii^—Na1—O2^iii^	90.07 (9)	O1^xxv^—Na4—Al1^xxx^	69.23 (15)
O1—Na1—O2^iii^	90.04 (9)	O1^xxvi^—Na4—Al1^xxx^	34.80 (7)
Na2^i^—Na1—O2^iv^	67.19 (14)	Na2^xxxix^—Na4—Al1^xxx^	74.23 (8)
O1^ii^—Na1—O2^iv^	90.04 (9)	Na2^xiv^—Na4—Al1^xxx^	74.23 (8)
O1—Na1—O2^iv^	90.07 (9)	Na2^xl^—Na4—Al1^xxx^	175.4 (4)
O2^iii^—Na1—O2^iv^	134.4 (3)	Cl2—Na4—Al1^xxx^	115.28 (18)
Na2^i^—Na1—Na3^v^	123.9 (3)	O2^xxvii^—Na4—Al1^xxx^	33.88 (5)
O1^ii^—Na1—Na3^v^	74.16 (9)	O2^xxviii^—Na4—Al1^xxx^	130.86 (15)
O1—Na1—Na3^v^	105.68 (13)	O2^xxix^—Na4—Al1^xxx^	108.51 (9)
O2^iii^—Na1—Na3^v^	59.5 (2)	O1^xxiv^—Na4—Al1^xxxi^	34.80 (7)
O2^iv^—Na1—Na3^v^	159.92 (19)	O1^xxv^—Na4—Al1^xxxi^	118.0 (4)
Na2^i^—Na1—Na3^vi^	123.9 (3)	O1^xxvi^—Na4—Al1^xxxi^	69.23 (15)
O1^ii^—Na1—Na3^vi^	105.68 (13)	Na2^xxxix^—Na4—Al1^xxxi^	175.4 (4)
O1—Na1—Na3^vi^	74.16 (9)	Na2^xiv^—Na4—Al1^xxxi^	74.23 (8)
O2^iii^—Na1—Na3^vi^	159.92 (19)	Na2^xl^—Na4—Al1^xxxi^	74.23 (8)
O2^iv^—Na1—Na3^vi^	59.5 (2)	Cl2—Na4—Al1^xxxi^	115.28 (18)
Na3^v^—Na1—Na3^vi^	112.2 (5)	O2^xxvii^—Na4—Al1^xxxi^	130.86 (15)
Na2^i^—Na1—Cl1	180.0	O2^xxviii^—Na4—Al1^xxxi^	108.51 (9)
O1^ii^—Na1—Cl1	89.86 (17)	O2^xxix^—Na4—Al1^xxxi^	33.88 (5)
O1—Na1—Cl1	89.86 (17)	Al1^xxx^—Na4—Al1^xxxi^	103.1 (2)
O2^iii^—Na1—Cl1	112.81 (14)	O1^xxxii^—B1—O1^xxxiii^	119.995 (7)
O2^iv^—Na1—Cl1	112.81 (14)	O1^xxxii^—B1—O1	119.995 (7)
Na3^v^—Na1—Cl1	56.1 (3)	O1^xxxiii^—B1—O1	119.992 (7)
Na3^vi^—Na1—Cl1	56.1 (3)	O1^xxxii^—B1—Na1^xxxii^	46.89 (18)
Na2^i^—Na1—B1^ii^	108.51 (15)	O1^xxxiii^—B1—Na1^xxxii^	135.4 (3)
O1^ii^—Na1—B1^ii^	26.25 (9)	O1—B1—Na1^xxxii^	87.58 (17)
O1—Na1—B1^ii^	153.5 (2)	O1^xxxii^—B1—Na1^xxxiii^	87.58 (17)
O2^iii^—Na1—B1^ii^	114.16 (5)	O1^xxxiii^—B1—Na1^xxxiii^	46.89 (18)
O2^iv^—Na1—B1^ii^	80.61 (6)	O1—B1—Na1^xxxiii^	135.4 (3)
Na3^v^—Na1—B1^ii^	79.81 (13)	Na1^xxxii^—B1—Na1^xxxiii^	88.6 (2)
Na3^vi^—Na1—B1^ii^	79.81 (13)	O1^xxxii^—B1—Na1	135.4 (3)
Cl1—Na1—B1^ii^	71.49 (15)	O1^xxxiii^—B1—Na1	87.58 (17)
Na2^i^—Na1—B1	108.51 (15)	O1—B1—Na1	46.89 (18)
O1^ii^—Na1—B1	153.5 (2)	Na1^xxxii^—B1—Na1	88.6 (2)
O1—Na1—B1	26.25 (9)	Na1^xxxiii^—B1—Na1	88.6 (2)
O2^iii^—Na1—B1	80.61 (6)	O2^xxxiv^—B2—O2^xxxv^	119.993 (8)
O2^iv^—Na1—B1	114.16 (5)	O2^xxxiv^—B2—O2^x^	119.994 (8)
Na3^v^—Na1—B1	79.81 (13)	O2^xxxv^—B2—O2^x^	119.995 (8)
Na3^vi^—Na1—B1	79.81 (13)	O2^xxxiv^—B2—Na2	135.7 (3)
Cl1—Na1—B1	71.49 (15)	O2^xxxv^—B2—Na2	87.51 (17)
B1^ii^—Na1—B1	143.0 (3)	O2^x^—B2—Na2	46.65 (18)
Na2^i^—Na1—Na4^vii^	42.1 (2)	O2^xxxiv^—B2—Na2^xxxiii^	46.65 (18)
O1^ii^—Na1—Na4^vii^	129.4 (3)	O2^xxxv^—B2—Na2^xxxiii^	135.7 (3)
O1—Na1—Na4^vii^	50.85 (19)	O2^x^—B2—Na2^xxxiii^	87.51 (17)
O2^iii^—Na1—Na4^vii^	60.65 (11)	Na2—B2—Na2^xxxiii^	89.2 (2)
O2^iv^—Na1—Na4^vii^	85.09 (18)	O2^xxxiv^—B2—Na2^xxxii^	87.51 (17)
Na3^v^—Na1—Na4^vii^	114.45 (17)	O2^xxxv^—B2—Na2^xxxii^	46.65 (18)
Na3^vi^—Na1—Na4^vii^	114.45 (17)	O2^x^—B2—Na2^xxxii^	135.7 (3)
Cl1—Na1—Na4^vii^	137.9 (2)	Na2—B2—Na2^xxxii^	89.2 (2)
B1^ii^—Na1—Na4^vii^	150.6 (3)	Na2^xxxiii^—B2—Na2^xxxii^	89.2 (2)
B1—Na1—Na4^vii^	66.4 (2)	O2^xxxvi^—Al1—O2^xxxvii^	111.44 (14)
Na2^i^—Na1—Na4^viii^	42.1 (2)	O2^xxxvi^—Al1—O1^xxxviii^	108.50 (8)
O1^ii^—Na1—Na4^viii^	50.85 (19)	O2^xxxvii^—Al1—O1^xxxviii^	108.53 (8)
O1—Na1—Na4^viii^	129.4 (3)	O2^xxxvi^—Al1—O1^xxxii^	108.53 (8)
O2^iii^—Na1—Na4^viii^	85.09 (18)	O2^xxxvii^—Al1—O1^xxxii^	108.50 (8)
O2^iv^—Na1—Na4^viii^	60.65 (11)	O1^xxxviii^—Al1—O1^xxxii^	111.37 (14)
Na3^v^—Na1—Na4^viii^	114.45 (17)	O2^xxxvi^—Al1—Na4^xli^	157.72 (8)
Na3^vi^—Na1—Na4^viii^	114.45 (17)	O2^xxxvii^—Al1—Na4^xli^	68.19 (18)
Cl1—Na1—Na4^viii^	137.9 (2)	O1^xxxviii^—Al1—Na4^xli^	92.00 (14)
B1^ii^—Na1—Na4^viii^	66.4 (2)	O1^xxxii^—Al1—Na4^xli^	53.90 (9)
B1—Na1—Na4^viii^	150.6 (3)	O2^xxxvi^—Al1—Na4^xlii^	68.19 (18)
Na4^vii^—Na1—Na4^viii^	84.1 (4)	O2^xxxvii^—Al1—Na4^xlii^	157.72 (8)
Na1^xii^—Na2—O2^ix^	90.71 (17)	O1^xxxviii^—Al1—Na4^xlii^	53.90 (9)
Na1^xii^—Na2—O2^x^	90.71 (17)	O1^xxxii^—Al1—Na4^xlii^	92.00 (14)
O2^ix^—Na2—O2^x^	178.6 (3)	Na4^xli^—Al1—Na4^xlii^	121.1 (4)
Na1^xii^—Na2—O1^xi^	67.66 (14)	O2^xxxvi^—Al1—Na3^ii^	54.03 (9)
O2^ix^—Na2—O1^xi^	90.25 (9)	O2^xxxvii^—Al1—Na3^ii^	92.18 (13)
O2^x^—Na2—O1^xi^	90.29 (9)	O1^xxxviii^—Al1—Na3^ii^	157.61 (8)
Na1^xii^—Na2—O1^xii^	67.66 (15)	O1^xxxii^—Al1—Na3^ii^	67.99 (17)
O2^ix^—Na2—O1^xii^	90.29 (9)	Na4^xli^—Al1—Na3^ii^	103.88 (11)
O2^x^—Na2—O1^xii^	90.25 (9)	Na4^xlii^—Al1—Na3^ii^	103.88 (11)
O1^xi^—Na2—O1^xii^	135.3 (3)	O2^xxxvi^—Al1—Na3^xliii^	92.18 (13)
Na1^xii^—Na2—Na4^xiii^	124.0 (3)	O2^xxxvii^—Al1—Na3^xliii^	54.03 (9)
O2^ix^—Na2—Na4^xiii^	105.33 (14)	O1^xxxviii^—Al1—Na3^xliii^	67.99 (17)
O2^x^—Na2—Na4^xiii^	73.84 (9)	O1^xxxii^—Al1—Na3^xliii^	157.61 (8)
O1^xi^—Na2—Na4^xiii^	159.6 (2)	Na4^xli^—Al1—Na3^xliii^	103.88 (11)
O1^xii^—Na2—Na4^xiii^	59.2 (2)	Na4^xlii^—Al1—Na3^xliii^	103.88 (11)
Na1^xii^—Na2—Na4^xiv^	124.0 (3)	Na3^ii^—Al1—Na3^xliii^	121.6 (4)
O2^ix^—Na2—Na4^xiv^	73.84 (9)	O2^xxxvi^—Al1—Na2^xliv^	68.10 (10)
O2^x^—Na2—Na4^xiv^	105.33 (14)	O2^xxxvii^—Al1—Na2^xliv^	111.93 (11)
O1^xi^—Na2—Na4^xiv^	59.2 (2)	O1^xxxviii^—Al1—Na2^xliv^	42.50 (8)
O1^xii^—Na2—Na4^xiv^	159.6 (2)	O1^xxxii^—Al1—Na2^xliv^	137.46 (9)
Na4^xiii^—Na2—Na4^xiv^	111.9 (5)	Na4^xli^—Al1—Na2^xliv^	133.72 (12)
Na1^xii^—Na2—Cl2	180.0	Na4^xlii^—Al1—Na2^xliv^	46.26 (12)
O2^ix^—Na2—Cl2	89.29 (17)	Na3^ii^—Al1—Na2^xliv^	122.08 (10)
O2^x^—Na2—Cl2	89.29 (17)	Na3^xliii^—Al1—Na2^xliv^	57.94 (10)
O1^xi^—Na2—Cl2	112.34 (14)	O2^xxxvi^—Al1—Na2^xlv^	36.03 (6)
O1^xii^—Na2—Cl2	112.34 (14)	O2^xxxvii^—Al1—Na2^xlv^	144.01 (8)
Na4^xiii^—Na2—Cl2	56.0 (3)	O1^xxxviii^—Al1—Na2^xlv^	99.67 (11)
Na4^xiv^—Na2—Cl2	56.0 (3)	O1^xxxii^—Al1—Na2^xlv^	80.31 (10)
Na1^xii^—Na2—B2^xv^	108.90 (15)	Na4^xli^—Al1—Na2^xlv^	133.72 (12)
O2^ix^—Na2—B2^xv^	26.12 (9)	Na4^xlii^—Al1—Na2^xlv^	46.26 (12)
O2^x^—Na2—B2^xv^	152.8 (2)	Na3^ii^—Al1—Na2^xlv^	57.94 (10)
O1^xi^—Na2—B2^xv^	114.17 (5)	Na3^xliii^—Al1—Na2^xlv^	122.08 (10)
O1^xii^—Na2—B2^xv^	80.61 (6)	Na2^xliv^—Al1—Na2^xlv^	74.9 (2)
Na4^xiii^—Na2—B2^xv^	79.56 (13)	O2^xxxvi^—Al1—Na2^xlvi^	144.01 (8)
Na4^xiv^—Na2—B2^xv^	79.56 (13)	O2^xxxvii^—Al1—Na2^xlvi^	36.03 (6)
Cl2—Na2—B2^xv^	71.10 (15)	O1^xxxviii^—Al1—Na2^xlvi^	80.31 (10)
Na1^xii^—Na2—B2	108.90 (15)	O1^xxxii^—Al1—Na2^xlvi^	99.67 (11)
O2^ix^—Na2—B2	152.9 (2)	Na4^xli^—Al1—Na2^xlvi^	46.26 (12)
O2^x^—Na2—B2	26.12 (9)	Na4^xlii^—Al1—Na2^xlvi^	133.72 (12)
O1^xi^—Na2—B2	80.61 (6)	Na3^ii^—Al1—Na2^xlvi^	122.08 (10)
O1^xii^—Na2—B2	114.17 (5)	Na3^xliii^—Al1—Na2^xlvi^	57.94 (10)
Na4^xiii^—Na2—B2	79.56 (13)	Na2^xliv^—Al1—Na2^xlvi^	105.1 (2)
Na4^xiv^—Na2—B2	79.56 (13)	Na2^xlv^—Al1—Na2^xlvi^	179.96 (4)
Cl2—Na2—B2	71.10 (15)	O2^xxxvi^—Al1—Na2^xlvii^	111.93 (11)
B2^xv^—Na2—B2	142.2 (3)	O2^xxxvii^—Al1—Na2^xlvii^	68.10 (10)
Na1^xii^—Na2—Na3^xvi^	42.1 (2)	O1^xxxviii^—Al1—Na2^xlvii^	137.46 (9)
O2^ix^—Na2—Na3^xvi^	130.0 (3)	O1^xxxii^—Al1—Na2^xlvii^	42.50 (8)
O2^x^—Na2—Na3^xvi^	51.37 (19)	Na4^xli^—Al1—Na2^xlvii^	46.26 (12)
O1^xi^—Na2—Na3^xvi^	61.00 (11)	Na4^xlii^—Al1—Na2^xlvii^	133.72 (12)
O1^xii^—Na2—Na3^xvi^	85.47 (18)	Na3^ii^—Al1—Na2^xlvii^	57.94 (10)
Na4^xiii^—Na2—Na3^xvi^	114.53 (18)	Na3^xliii^—Al1—Na2^xlvii^	122.08 (10)
Na4^xiv^—Na2—Na3^xvi^	114.53 (18)	Na2^xliv^—Al1—Na2^xlvii^	179.96 (12)
Cl2—Na2—Na3^xvi^	137.9 (2)	Na2^xlv^—Al1—Na2^xlvii^	105.1 (2)
B2^xv^—Na2—Na3^xvi^	151.0 (3)	Na2^xlvi^—Al1—Na2^xlvii^	74.9 (2)
B2—Na2—Na3^xvi^	66.8 (2)	B1—O1—Al1^xxxiii^	128.62 (14)
Na1^xii^—Na2—Na3^xvii^	42.1 (2)	B1—O1—Na1	106.9 (3)
O2^ix^—Na2—Na3^xvii^	51.37 (18)	Al1^xxxiii^—O1—Na1	117.02 (10)
O2^x^—Na2—Na3^xvii^	130.0 (3)	B1—O1—Na2^i^	121.75 (19)
O1^xi^—Na2—Na3^xvii^	85.47 (18)	Al1^xxxiii^—O1—Na2^i^	108.49 (10)
O1^xii^—Na2—Na3^xvii^	61.00 (11)	Na1—O1—Na2^i^	22.20 (14)
Na4^xiii^—Na2—Na3^xvii^	114.53 (18)	B1—O1—Na4^vii^	119.4 (2)
Na4^xiv^—Na2—Na3^xvii^	114.53 (18)	Al1^xxxiii^—O1—Na4^vii^	91.30 (7)
Cl2—Na2—Na3^xvii^	137.9 (2)	Na1—O1—Na4^vii^	84.2 (2)
B2^xv^—Na2—Na3^xvii^	66.8 (2)	Na2^i^—O1—Na4^vii^	63.0 (2)
B2—Na2—Na3^xvii^	151.0 (3)	B1—O1—Na3^vi^	106.5 (3)
Na3^xvi^—Na2—Na3^xvii^	84.2 (4)	Al1^xxxiii^—O1—Na3^vi^	78.1 (2)
O2^xviii^—Na3—O2^xix^	92.1 (3)	Na1—O1—Na3^vi^	57.59 (12)
O2^xviii^—Na3—O2^xx^	92.1 (3)	Na2^i^—O1—Na3^vi^	71.84 (11)
O2^xix^—Na3—O2^xx^	92.1 (3)	Na4^vii^—O1—Na3^vi^	127.3 (2)
O2^xviii^—Na3—Na1^vi^	143.25 (16)	B2^xlviii^—O2—Al1^xxii^	128.66 (14)
O2^xix^—Na3—Na1^vi^	108.65 (10)	B2^xlviii^—O2—Na2^xlviii^	107.2 (3)
O2^xx^—Na3—Na1^vi^	58.15 (12)	Al1^xxii^—O2—Na2^xlviii^	116.89 (10)
O2^xviii^—Na3—Na1^xxi^	58.15 (12)	B2^xlviii^—O2—Na1^xlix^	121.99 (19)
O2^xix^—Na3—Na1^xxi^	143.26 (16)	Al1^xxii^—O2—Na1^xlix^	108.31 (10)
O2^xx^—Na3—Na1^xxi^	108.65 (10)	Na2^xlviii^—O2—Na1^xlix^	22.10 (14)
Na1^vi^—Na3—Na1^xxi^	108.1 (3)	B2^xlviii^—O2—Na3^l^	119.5 (2)
O2^xviii^—Na3—Na1^xxii^	108.65 (10)	Al1^xxii^—O2—Na3^l^	91.29 (7)
O2^xix^—Na3—Na1^xxii^	58.15 (12)	Na2^xlviii^—O2—Na3^l^	83.4 (2)
O2^xx^—Na3—Na1^xxii^	143.25 (16)	Na1^xlix^—O2—Na3^l^	62.3 (2)
Na1^vi^—Na3—Na1^xxii^	108.1 (3)	B2^xlviii^—O2—Na4^li^	106.8 (3)
Na1^xxi^—Na3—Na1^xxii^	108.1 (3)	Al1^xxii^—O2—Na4^li^	77.9 (2)
O2^xviii^—Na3—Cl1	123.8 (2)	Na2^xlviii^—O2—Na4^li^	58.00 (12)
O2^xix^—Na3—Cl1	123.8 (2)	Na1^xlix^—O2—Na4^li^	72.17 (10)
O2^xx^—Na3—Cl1	123.8 (2)	Na3^l^—O2—Na4^li^	126.9 (2)
Na1^vi^—Na3—Cl1	69.1 (3)	Na3—Cl1—Na3^ii^	109.472 (1)
Na1^xxi^—Na3—Cl1	69.1 (3)	Na3—Cl1—Na3^v^	109.5
Na1^xxii^—Na3—Cl1	69.1 (3)	Na3^ii^—Cl1—Na3^v^	109.5
O2^xviii^—Na3—O1^xxi^	75.84 (8)	Na3—Cl1—Na3^vi^	109.5
O2^xix^—Na3—O1^xxi^	151.3 (4)	Na3^ii^—Cl1—Na3^vi^	109.5
O2^xx^—Na3—O1^xxi^	62.94 (6)	Na3^v^—Cl1—Na3^vi^	109.5
Na1^vi^—Na3—O1^xxi^	71.52 (7)	Na3—Cl1—Na1^vi^	54.7
Na1^xxi^—Na3—O1^xxi^	48.25 (5)	Na3^ii^—Cl1—Na1^vi^	54.736 (1)
Na1^xxii^—Na3—O1^xxi^	150.4 (4)	Na3^v^—Cl1—Na1^vi^	125.3
Cl1—Na3—O1^xxi^	83.9 (2)	Na3^vi^—Cl1—Na1^vi^	125.3
O2^xviii^—Na3—O1^xxii^	62.94 (6)	Na3—Cl1—Na1^xxii^	54.7
O2^xix^—Na3—O1^xxii^	75.84 (8)	Na3^ii^—Cl1—Na1^xxii^	125.3
O2^xx^—Na3—O1^xxii^	151.3 (4)	Na3^v^—Cl1—Na1^xxii^	54.7
Na1^vi^—Na3—O1^xxii^	150.4 (4)	Na3^vi^—Cl1—Na1^xxii^	125.3
Na1^xxi^—Na3—O1^xxii^	71.53 (7)	Na1^vi^—Cl1—Na1^xxii^	90.0
Na1^xxii^—Na3—O1^xxii^	48.25 (5)	Na3—Cl1—Na1^xxxiii^	125.3
Cl1—Na3—O1^xxii^	83.9 (2)	Na3^ii^—Cl1—Na1^xxxiii^	54.7
O1^xxi^—Na3—O1^xxii^	118.89 (7)	Na3^v^—Cl1—Na1^xxxiii^	54.7
O2^xviii^—Na3—O1^vi^	151.3 (4)	Na3^vi^—Cl1—Na1^xxxiii^	125.3
O2^xix^—Na3—O1^vi^	62.94 (6)	Na1^vi^—Cl1—Na1^xxxiii^	90.0
O2^xx^—Na3—O1^vi^	75.84 (8)	Na1^xxii^—Cl1—Na1^xxxiii^	90.0
Na1^vi^—Na3—O1^vi^	48.25 (5)	Na3—Cl1—Na1^xxi^	54.7
Na1^xxi^—Na3—O1^vi^	150.4 (4)	Na3^ii^—Cl1—Na1^xxi^	125.3
Na1^xxii^—Na3—O1^vi^	71.52 (7)	Na3^v^—Cl1—Na1^xxi^	125.3
Cl1—Na3—O1^vi^	83.9 (2)	Na3^vi^—Cl1—Na1^xxi^	54.7
O1^xxi^—Na3—O1^vi^	118.88 (7)	Na1^vi^—Cl1—Na1^xxi^	90.0
O1^xxii^—Na3—O1^vi^	118.88 (7)	Na1^xxii^—Cl1—Na1^xxi^	90.0
O2^xviii^—Na3—Al1^xvii^	34.68 (7)	Na1^xxxiii^—Cl1—Na1^xxi^	180.0
O2^xix^—Na3—Al1^xvii^	69.06 (15)	Na3—Cl1—Na1^xxxii^	125.3
O2^xx^—Na3—Al1^xvii^	117.4 (4)	Na3^ii^—Cl1—Na1^xxxii^	54.7
Na1^vi^—Na3—Al1^xvii^	175.3 (4)	Na3^v^—Cl1—Na1^xxxii^	125.3
Na1^xxi^—Na3—Al1^xvii^	74.45 (8)	Na3^vi^—Cl1—Na1^xxxii^	54.7
Na1^xxii^—Na3—Al1^xvii^	74.45 (8)	Na1^vi^—Cl1—Na1^xxxii^	90.0
Cl1—Na3—Al1^xvii^	115.54 (18)	Na1^xxii^—Cl1—Na1^xxxii^	180.0
O1^xxi^—Na3—Al1^xvii^	108.38 (9)	Na1^xxxiii^—Cl1—Na1^xxxii^	90.0
O1^xxii^—Na3—Al1^xvii^	33.91 (5)	Na1^xxi^—Cl1—Na1^xxxii^	90.0
O1^vi^—Na3—Al1^xvii^	130.71 (15)	Na3—Cl1—Na1	125.3
O2^xviii^—Na3—Al1^xxiii^	117.4 (4)	Na3^ii^—Cl1—Na1	125.264 (1)
O2^xix^—Na3—Al1^xxiii^	34.68 (7)	Na3^v^—Cl1—Na1	54.7
O2^xx^—Na3—Al1^xxiii^	69.06 (15)	Na3^vi^—Cl1—Na1	54.7
Na1^vi^—Na3—Al1^xxiii^	74.45 (8)	Na1^vi^—Cl1—Na1	180.0
Na1^xxi^—Na3—Al1^xxiii^	175.3 (4)	Na1^xxii^—Cl1—Na1	90.0
Na1^xxii^—Na3—Al1^xxiii^	74.45 (8)	Na1^xxxiii^—Cl1—Na1	90.0
Cl1—Na3—Al1^xxiii^	115.54 (18)	Na1^xxi^—Cl1—Na1	90.0
O1^xxi^—Na3—Al1^xxiii^	130.71 (14)	Na1^xxxii^—Cl1—Na1	90.0
O1^xxii^—Na3—Al1^xxiii^	108.38 (10)	Na4^xiv^—Cl2—Na4^xv^	109.472 (1)
O1^vi^—Na3—Al1^xxiii^	33.91 (5)	Na4^xiv^—Cl2—Na4^xiii^	109.470 (1)
Al1^xvii^—Na3—Al1^xxiii^	102.8 (2)	Na4^xv^—Cl2—Na4^xiii^	109.472 (1)
O1^xxiv^—Na4—O1^xxv^	92.5 (3)	Na4^xiv^—Cl2—Na4	109.5
O1^xxiv^—Na4—O1^xxvi^	92.5 (3)	Na4^xv^—Cl2—Na4	109.470 (2)
O1^xxv^—Na4—O1^xxvi^	92.5 (3)	Na4^xiii^—Cl2—Na4	109.472 (1)
O1^xxiv^—Na4—Na2^xxxix^	143.20 (16)	Na4^xiv^—Cl2—Na2^xxxii^	54.7
O1^xxv^—Na4—Na2^xxxix^	57.75 (12)	Na4^xv^—Cl2—Na2^xxxii^	54.7
O1^xxvi^—Na4—Na2^xxxix^	108.55 (10)	Na4^xiii^—Cl2—Na2^xxxii^	125.264 (2)
O1^xxiv^—Na4—Na2^xiv^	108.55 (10)	Na4—Cl2—Na2^xxxii^	125.3
O1^xxv^—Na4—Na2^xiv^	143.20 (16)	Na4^xiv^—Cl2—Na2^xxxix^	125.3
O1^xxvi^—Na4—Na2^xiv^	57.75 (12)	Na4^xv^—Cl2—Na2^xxxix^	125.264 (1)
Na2^xxxix^—Na4—Na2^xiv^	108.2 (3)	Na4^xiii^—Cl2—Na2^xxxix^	54.736 (1)
O1^xxiv^—Na4—Na2^xl^	57.75 (12)	Na4—Cl2—Na2^xxxix^	54.7
O1^xxv^—Na4—Na2^xl^	108.55 (10)	Na2^xxxii^—Cl2—Na2^xxxix^	180.0
O1^xxvi^—Na4—Na2^xl^	143.20 (16)	Na4^xiv^—Cl2—Na2^xxxiii^	125.264 (1)
Na2^xxxix^—Na4—Na2^xl^	108.2 (3)	Na4^xv^—Cl2—Na2^xxxiii^	54.736 (1)
Na2^xiv^—Na4—Na2^xl^	108.2 (3)	Na4^xiii^—Cl2—Na2^xxxiii^	54.7
O1^xxiv^—Na4—Cl2	123.5 (2)	Na4—Cl2—Na2^xxxiii^	125.264 (1)
O1^xxv^—Na4—Cl2	123.5 (2)	Na2^xxxii^—Cl2—Na2^xxxiii^	90.0
O1^xxvi^—Na4—Cl2	123.5 (2)	Na2^xxxix^—Cl2—Na2^xxxiii^	90.0
Na2^xxxix^—Na4—Cl2	69.3 (3)	Na4^xiv^—Cl2—Na2^xl^	54.736 (1)
Na2^xiv^—Na4—Cl2	69.3 (3)	Na4^xv^—Cl2—Na2^xl^	125.3
Na2^xl^—Na4—Cl2	69.3 (3)	Na4^xiii^—Cl2—Na2^xl^	125.264 (1)
O1^xxiv^—Na4—O2^xxvii^	151.8 (4)	Na4—Cl2—Na2^xl^	54.736 (1)
O1^xxv^—Na4—O2^xxvii^	75.89 (8)	Na2^xxxii^—Cl2—Na2^xl^	90.0
O1^xxvi^—Na4—O2^xxvii^	62.98 (6)	Na2^xxxix^—Cl2—Na2^xl^	90.0
Na2^xxxix^—Na4—O2^xxvii^	48.16 (5)	Na2^xxxiii^—Cl2—Na2^xl^	180.0
Na2^xiv^—Na4—O2^xxvii^	71.42 (7)	Na4^xiv^—Cl2—Na2	54.735 (1)
Na2^xl^—Na4—O2^xxvii^	150.3 (4)	Na4^xv^—Cl2—Na2	125.265 (1)
Cl2—Na4—O2^xxvii^	83.7 (2)	Na4^xiii^—Cl2—Na2	54.735 (1)
O1^xxiv^—Na4—O2^xxviii^	75.89 (8)	Na4—Cl2—Na2	125.3
O1^xxv^—Na4—O2^xxviii^	62.98 (6)	Na2^xxxii^—Cl2—Na2	90.000 (2)
O1^xxvi^—Na4—O2^xxviii^	151.8 (4)	Na2^xxxix^—Cl2—Na2	90.0
Na2^xxxix^—Na4—O2^xxviii^	71.42 (7)	Na2^xxxiii^—Cl2—Na2	90.000 (1)
Na2^xiv^—Na4—O2^xxviii^	150.3 (4)	Na2^xl^—Cl2—Na2	90.0
Na2^xl^—Na4—O2^xxviii^	48.16 (5)	Na4^xiv^—Cl2—Na2^xiv^	125.3
Cl2—Na4—O2^xxviii^	83.7 (2)	Na4^xv^—Cl2—Na2^xiv^	54.735 (1)
O2^xxvii^—Na4—O2^xxviii^	118.80 (8)	Na4^xiii^—Cl2—Na2^xiv^	125.265 (1)
O1^xxiv^—Na4—O2^xxix^	62.98 (6)	Na4—Cl2—Na2^xiv^	54.735 (1)
O1^xxv^—Na4—O2^xxix^	151.8 (4)	Na2^xxxii^—Cl2—Na2^xiv^	90.0
O1^xxvi^—Na4—O2^xxix^	75.89 (8)	Na2^xxxix^—Cl2—Na2^xiv^	90.000 (2)
Na2^xxxix^—Na4—O2^xxix^	150.3 (4)	Na2^xxxiii^—Cl2—Na2^xiv^	90.0
Na2^xiv^—Na4—O2^xxix^	48.16 (5)	Na2^xl^—Cl2—Na2^xiv^	90.000 (1)
Na2^xl^—Na4—O2^xxix^	71.42 (7)	Na2—Cl2—Na2^xiv^	180.0

Symmetry codes: (i) −*x*+1/2, −*y*+1, *z*−1/2; (ii) *x*, −*y*+1/2, −*z*+1/2; (iii) −*x*, −*y*+1/2, *z*−1/2; (iv) −*x*, *y*, −*z*+1; (v) −*x*+1/2, *y*, −*z*+1/2; (vi) −*x*+1/2, −*y*+1/2, *z*; (vii) *x*−1, −*y*+1, −*z*+1; (viii) *x*−1, *y*−1/2, *z*−1/2; (ix) *x*+1/2, −*y*+1, −*z*+3/2; (x) *x*+1/2, *y*+1/2, *z*; (xi) −*x*+1/2, *y*+1/2, −*z*+1; (xii) −*x*+1/2, −*y*+1, *z*+1/2; (xiii) −*x*+3/2, *y*, −*z*+3/2; (xiv) −*x*+3/2, −*y*+3/2, *z*; (xv) *x*, −*y*+3/2, −*z*+3/2; (xvi) *x*, −*y*+1, −*z*+1; (xvii) *x*, *y*+1/2, *z*+1/2; (xviii) −*y*+1/2, −*z*+1, *x*+1/2; (xix) −*z*+1, *x*+1/2, −*y*+1/2; (xx) *x*+1/2, −*y*+1/2, −*z*+1; (xxi) −*y*+1/2, *z*, −*x*+1/2; (xxii) *z*, −*x*+1/2, −*y*+1/2; (xxiii) *y*+1/2, *z*+1/2, *x*; (xxiv) −*y*+1, −*z*+1, *x*+1; (xxv) −*z*+1, *x*+1, −*y*+1; (xxvi) *x*+1, −*y*+1, −*z*+1; (xxvii) −*z*+3/2, −*x*+1, *y*+1/2; (xxviii) *y*+1/2, −*z*+3/2, −*x*+1; (xxix) −*x*+1, *y*+1/2, −*z*+3/2; (xxx) −*y*+1, *z*+1, −*x*+1; (xxxi) *z*+1, −*x*+1, −*y*+1; (xxxii) *z*, *x*, *y*; (xxxiii) *y*, *z*, *x*; (xxxiv) *y*+1/2, *z*, *x*+1/2; (xxxv) *z*, *x*+1/2, *y*+1/2; (xxxvi) −*y*+1/2, *z*−1/2, −*x*; (xxxvii) −*y*+1/2, −*z*+1/2, *x*; (xxxviii) *z*, −*x*, −*y*; (xxxix) *z*, −*x*+3/2, −*y*+3/2; (xl) −*y*+3/2, *z*, −*x*+3/2; (xli) −*x*+1, *y*−1, −*z*+1; (xlii) −*x*+1, −*y*+1, *z*−1; (xliii) *x*, *y*−1/2, *z*−1/2; (xliv) *z*−1/2, *x*−1/2, *y*−1; (xlv) −*y*+1, *z*−1/2, −*x*+1/2; (xlvi) *y*−1/2, *z*−1, *x*−1/2; (xlvii) *z*−1/2, −*x*+1/2, −*y*+1; (xlviii) *x*−1/2, *y*−1/2, *z*; (xlix) −*x*, −*y*+1/2, *z*+1/2; (l) *x*−1/2, −*y*+1/2, −*z*+1; (li) −*x*+1, *y*−1/2, −*z*+3/2.

## References

[bb1] Bruker (2019). *SAINT*. Bruker AXS Inc., Madison, Wisconsin, USA.

[bb2] Bruker (2021). *BIS*. Bruker AXS Inc., Madison, Wisconsin, USA.

[bb3] Calès, B., Levasseur, A., Fouassier, C., Réau, J. M. & Hagenmuller, P. (1977). *Solid State Commun.* **24**, 323–325.

[bb4] Farrugia, L. J. (2012). *J. Appl. Cryst.* **45**, 849–854.

[bb5] Hübschle, C. B., Sheldrick, G. M. & Dittrich, B. (2011). *J. Appl. Cryst.* **44**, 1281–1284. 10.1107/S0021889811043202 PMC3246833 22477785

[bb6] Jeitschko, W., Bither, T. A. & Bierstedt, P. E. (1977). *Acta Cryst.* B**33**, 2767–2775.

[bb7] Kajihara, K., Tezuka, N., Shoji, M., Wakasugi, J., Munakata, H. & Kanamura, K. (2017). *Bull. Chem. Soc. Jpn*, **90**, 1279–1286.

[bb8] Katsumata, T., Aoki, Y., Fushimi, K., Otsuka, K., Ueda, K. & Inaguma, Y. (2022). *Solid State Ionics*, **380**, 115921.

[bb9] Kaup, K., Bishop, K., Assoud, A., Liu, J. & Nazar, L. F. (2021). *J. Am. Chem. Soc.* **143**, 6952–6961. 10.1021/jacs.1c00941 33929830

[bb10] Krause, L., Herbst-Irmer, R., Sheldrick, G. M. & Stalke, D. (2015). *J. Appl. Cryst.* **48**, 3–10. 10.1107/S1600576714022985 PMC4453166 26089746

[bb11] Levasseur, A., Fouassier, C. & Hagenmuller, P. (1971). *Mater. Res. Bull.* **6**, 15–22.

[bb12] Li, Y. & Holzwarth, N. A. W. (2022). *Phys. Rev. Mater.* **6**, 025401.

[bb13] Momma, K. & Izumi, F. (2011). *J. Appl. Cryst.* **44**, 1272–1276.

[bb14] Nelmes, R. J. (1974). *J. Phys. C.: Solid State Phys.* **7**, 3840–3854.

[bb15] Parsons, S., Flack, H. D. & Wagner, T. (2013). *Acta Cryst.* B**69**, 249–259. 10.1107/S2052519213010014 PMC3661305 23719469

[bb16] Réau, J.-M., Levasseur, A., Magniez, G. & Calès, B. (1976). *Mater. Res. Bull.* **11**, 1087–1090.

[bb17] Saito, M., Arima, H., Shoji, M., Kizuki, Y., Munakata, H., Kanamura, K. & Kajihara, K. (2021). *J. Electrochem. Soc.* **168**, 040524.

[bb18] Schmid, H. (1965). *J. Phys. Chem. Solids*, **26**, 973–976.

[bb19] Shannon, R. D. (1976). *Acta Cryst.* A**32**, 751–767.

[bb20] Sheldrick, G. M. (2015*a*). *Acta Cryst.* A**71**, 3–8.

[bb21] Sheldrick, G. M. (2015*b*). *Acta Cryst.* C**71**, 3–8.

[bb22] Sorokin, N. I. (2015). *Phys. Solid State*, **57**, 314–315.

[bb23] Tezuka, N., Okawa, Y., Kajihara, K. & Kanamura, K. (2017). *J. Ceram. Soc. Japan*, **125**, 348–352.

[bb24] Vlasse, M., Levasseur, A. & Hagenmuller, P. (1981). *Solid State Ionics*, **2**, 33–37.

[bb25] Westrip, S. P. (2010). *J. Appl. Cryst.* **43**, 920–925.

